# SbNAC9 Improves Drought Tolerance by Enhancing Scavenging Ability of Reactive Oxygen Species and Activating Stress-Responsive Genes of Sorghum

**DOI:** 10.3390/ijms24032401

**Published:** 2023-01-26

**Authors:** Xueying Jin, Yuchen Zheng, Jingyi Wang, Wei Chen, Zhen Yang, Yaxin Chen, Yonghua Yang, Guihua Lu, Bo Sun

**Affiliations:** State Key Laboratory of Pharmaceutical Biotechnology, School of Life Sciences, Nanjing University, Nanjing 210023, China

**Keywords:** *Sorghum bicolor*, drought stress, NAC transcription factor, reactive oxygen species (ROS), virus induced-gene silencing

## Abstract

Drought stress severely threatens the yield of cereal crops. Therefore, understanding the molecular mechanism of drought stress response of plants is crucial for developing drought-tolerant cultivars. NAC transcription factors (TFs) play important roles in abiotic stress of plants, but the functions of NAC TFs in sorghum are largely unknown. Here, we characterized a sorghum NAC gene, *SbNAC9*, and found that *SbNAC9* can be highly induced by polyethylene glycol (PEG)-simulated dehydration treatments. We therefore investigated the function of SbNAC9 in drought stress response. Sorghum seedlings overexpressing *SbNAC9* showed enhanced drought-stress tolerance with higher chlorophyll content and photochemical efficiency of PSII, stronger root systems, and higher reactive oxygen species (ROS) scavenging capability than wild-type. In contrast, sorghum seedlings with silenced *SbNAC9* by virus-induced gene silencing (VIGS) showed weakened drought stress tolerance. Furthermore, SbNAC9 can directly activate a putative peroxidase gene *SbC5YQ75* and a putative ABA biosynthesis gene *SbNCED3*. Silencing *SbC5YQ75* and *SbNCED3* led to compromised drought tolerance and reduced ABA content of sorghum seedlings, respectively. Therefore, our findings revealed the important role of SbNAC9 in response to drought stress in sorghum and may shed light on genetic improvement of other crop species under drought-stress conditions.

## 1. Introduction

Drought is one of the major environmental factors limiting the yield of cereal crops, and drought leads to a decrease in seed germination, retardation of vegetative development, and reduction of grain quantity and quality [1,2]. Sorghum (*Sorghum bicolor* (L.) Moench) is widely cultivated in arid and semiarid regions of developing countries and provides staple food for over 500 million people [3]. Although sorghum is generally tolerant to a drought environment, it is still susceptible to water scarcity at seedling stage, preflowering stage, and postflowering stage [3,4]. Water loss induced by drought stress may cause impaired cell division and elongation which may lead to severe inhibition of embryonic growth, accompanied with poor seedling establishment [2]. At postflowering stage, water deficiency directly influences grain size due to premature plant senescence [5]. Therefore, understanding the molecular mechanism of drought-stress tolerance of sorghum will be benefit to the yield of sorghum and even other cereal crops.

Drought stress can induce accumulation of reactive oxygen species (ROS) in plants, which include superoxide anions, hydroxyl radicals, hydrogen peroxide, and singlet oxygen [6]. ROS can harm molecular activities in cells and even may lead to programmed cell death [7,8]. Although low concentration of ROS is an important part of stress signaling [9], excess accumulation of ROS requires antioxidant enzymes such as superoxide dismutase (SOD), peroxidase (POD), and catalase (CAT) to eliminate ROS toxicity to cells [10,11]. ROS can also lead to an increase in lipid peroxidation in cell membranes, which can be indicated by malondialdehyde (MDA) content [12,13]. To cope with drought stress, plants adopt a series of physiological, morphological, and molecular strategies. There are abundant stress-responsive factors which can induce the protective enzyme activities to reduce ROS damage to cells.

The responses of plants to drought stress involve signaling cascades with a phytohormone ABA playing the central role [14]. For ABA biosynthesis, the small peptide CLAVATA3/EMBRYO-SURROUNDING REGIONRELATED 25 (CLE25) moves from the roots to the leaves to activate expression of the key enzyme gene *NINE-CIS-EPOXYCAROTENOID DIOXYGENASE 3* (*NCED3*) in Arabidopsis [15]. Induction of *NCED3* promotes rapid accumulation of ABA to trigger downstream signaling, which leads to stomatal closure to reduce water loss [16]. In addition to ABA signaling, transcription factors (TFs) also play vital roles under drought stress. In plants, NAC [no apical meristem (NAM), Arabidopsis transcription activation factor 1/2 (ATAF1/2), and cup-shaped cotyledon2 (CUC2)] family TFs [17,18], ethylene-responsive transcription factors, WRKY family TFs, heat stress transcription factors, and homeodomain leucine zipper family transcription factors all play important roles in drought-stress responses [3,19,20].

NAC TFs are one of the largest families of plant specific TFs. To date, 105 AtNAC TFs in Arabidopsis, 138 OsNAC TFs in rice, 131 SbNAC TFs in sorghum, and 147 ZmNAC TFs in maize have been identified [4,7,21,22]. Many NAC TFs are reported to be involved in abiotic stress responses in plants. NAC transcription factor *JUNGBRUNNEN1* (*JUB1*) from Arabidopsis played a positive role in regulating *Dehydration Responsive Element-Binding protein 1* (*SlDREB1*), *Dehydration Responsive Element-Binding protein 2* (*SlDREB2*), and *SlDELLA* in tomato under drought stress [23]. Overexpression of *OsNAC066* in rice upregulates the relative expression of stress-responsive genes including *Early-Responsive to Dehydration 1* (*OsERD1*), *Late Embryogenesis Abundant 3* (*OsLEA3*), and *Dehydration Responsive Element-Binding protein 2A* (*OsDREB2A*) [24]. Overexpression of *SlNAC6* in tomato leads to efficient closure of stomata to reduce water loss under PEG-simulated drought treatment [20]. Overexpression of *ZmNAC49* reduces not only stomatal conductance but also stomatal density to improve the plant stress tolerance in maize [22].

Although there are at least 131 NAC genes in sorghum, only a few SbNAC TFs have been functionally characterized [4]. *SbSNAC1* is highly similar to *OsSNAC1* of rice, which is responsive to multiple abiotic stresses and ABA treatment. Overexpression of *SbSNAC1* in Arabidopsis can enhance plant drought- and salt-stress tolerance [25]. SbNAC2 can directly activate stress responsive gene *SbAP37* and improve drought-stress tolerance of sorghum [26]. However, molecular functions of most sorghum SbNAC TFs remain to be elucidated.

In this study, we investigated the function of SbNAC9 and found that *SbNAC9* can respond to various abiotic stresses and ABA treatment, especially highly to PEG-simulated dehydration stress. We overexpressed *SbNAC9* in sorghum and found that the transgenic lines had enhanced plant drought-stress tolerance with increased photosynthesis, strengthened root architecture, and increased ROS scavenging ability. By contrast, in sorghum seedlings with virus-induced gene silencing (VIGS) of *SbNAC9*, drought-stress tolerance of the seedlings was obviously compromised. In addition, heterologous overexpression of *SbNAC9* could also enhance drought tolerance of Arabidopsis. Moreover, SbNAC9 could directly activate the expression of putative peroxidase gene *SbC5YQ75* [27] and putative enzyme gene *SbNCED3* for ABA biosynthesis. VIGS-mediated silencing of *SbC5YQ75* and *SbNCED3* led to weakened drought tolerance and reduced ABA content in sorghum seedlings, respectively. Overall, our findings revealed the detailed molecular mechanism of *SbNAC9* in response to drought stress in sorghum and shed light on genetic enhancement of sorghum and even other crop species.

## 2. Results

### 2.1. SbNAC9 Can Respond to PEG-Simulated Drought Stress

In a previous study, we constructed a phylogenetic tree based on 128 OsNAC TFs from rice and 119 SbNAC TFs from sorghum and noticed a subgroup containing 4 reported OsNAC TFs responsive to abiotic stress. Additionally, in this subgroup, we characterized a sorghum transcription factor, SbNAC2, which plays an important role under abiotic stresses [26]. However, the functions of other SbNAC TFs of this subgroup in abiotic stress need to be elucidated.

**Figure 1:**
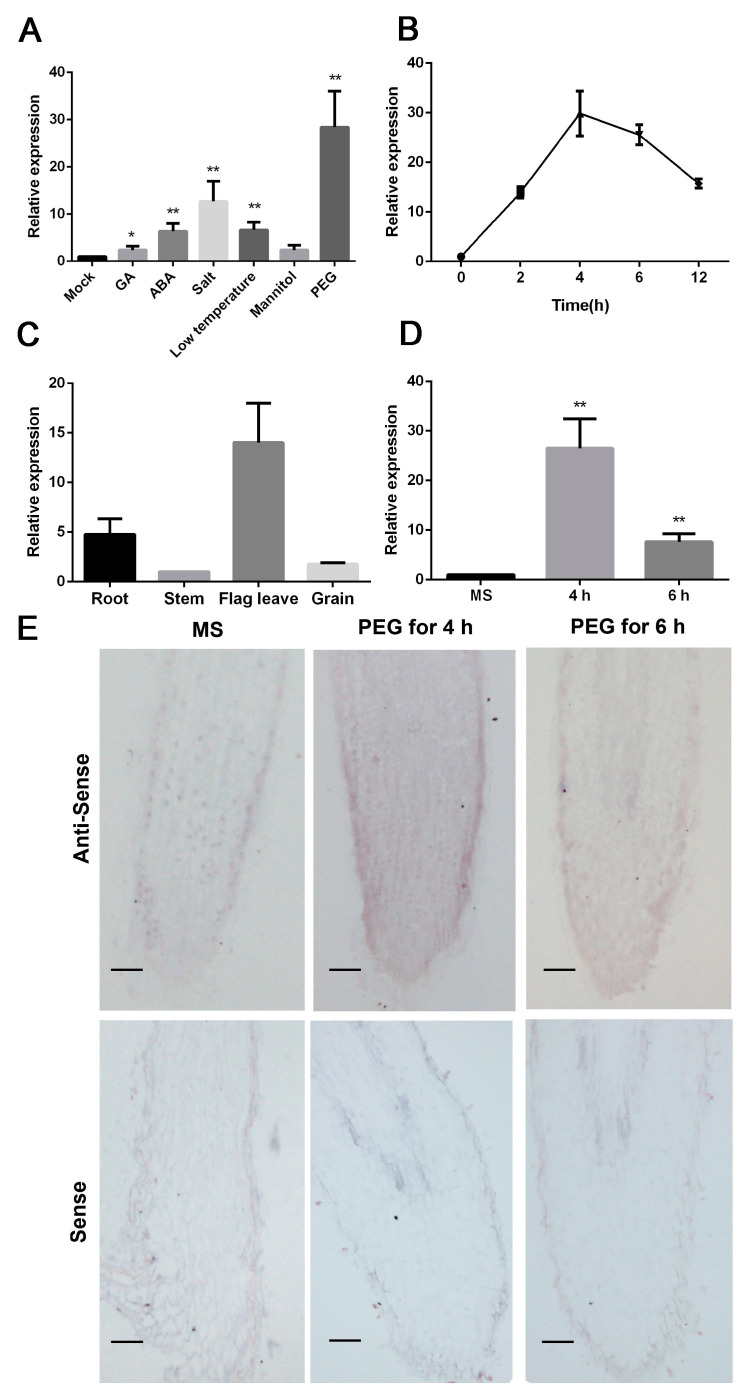
*SbNAC9* responds to PEG-simulated dehydration stress. (**A**) Relative expression of *SbNAC9* in the fourth leaf of sorghum seedlings at four-leaf stage under hormone and abiotic stress treatments. Sorghum seedlings were used for water (Mock), 150 μM GA (gibberellin), 150 μM ABA, 200 mM NaCl, 200 mM mannitol, and 20% PEG6000 treatments. For low temperature treatment, sorghum seedlings were kept at 4 °C. *SbEIF4A* was used as the internal reference. Error bars indicate SD of three independent experiments. ** *p* < 0.01 and * *p* < 0.05 by Student’s *t*-test. (**B**) Time course transcript level of *SbNAC9* in sorghum seedlings at four-leaf stage subjected to 20% PEG treatment. *SbEIF4A* was used as the internal reference. Error bars indicate SD of three independent experiments. (**C**) Relative transcript level of *SbNAC9* in various tissues of sorghum at filling stage. *SbEIF4A* was used as the internal reference. Error bars indicate SD of three independent experiments. (**D**) Relative expression level of *SbNAC9* in the three-day-old root tips of sorghum under MS media soaked into 20% PEG solution for 4 h and 6 h. *SbEIF4A* was used as the internal reference. Error bars indicate SD of three independent experiments. ** *p* < 0.01 by Student’s *t-*test. (**E**) In situ hybridization assay of *SbNAC9* in root tips of the three-day-old sorghum seedlings subjected to 20 % PEG-simulated drought-stress treatment for 4 h and 6 h. Root tips grown on MS media served as negative control. Bars indicate 100 μm.

Here, we chose the SOBIC.005G064600.2 (SbNAC9) which showed the highest protein sequence similarity with OsNAC5 [28] for subsequent study. First, we found that the *SbNAC9* promoter harbors many abiotic stress-related and hormone-responsive elements (Table S1), implying that *SbNAC9* may respond to abiotic stress. The transcript level of *SbNAC9* was upregulated under multiple abiotic stresses and phytohormone treatments. Among these treatments, *SbNAC9* had the highest expression under PEG-simulated dehydration treatment (Figure 1A). Therefore, we presumed that *SbNAC9* may play a potential role under drought stress in sorghum.

By PEG-simulated dehydration treatment, *SbNAC9* transcript could be obviously induced and peaked at 4 h (Figure 1B). Meanwhile, *SbNAC9* could also be induced and peaked at 1 h by ABA treatment (Figure S1). We also analyzed *SbNAC9* expression in various sorghum tissues under normal growth condition. In roots and leaves, *SbNAC9* transcript level was higher than in other tissues (Figure 1C). As roots may sense water deficiency in soil [29], we used sorghum root treated with 20% PEG6000 solution [25], which simulated water deficiency, for further analysis. After 4 h treatment, *SbNAC9* activity was obviously induced in roots as shown by both qRT-PCR (quantitative real-time PCR) and in situ hybridization results (Figure 1D,E). These results showed that *SbNAC9* can quickly respond to PEG-simulated drought stress and may play a potential role in drought-stress tolerance of sorghum.

### 2.2. SbNAC9 Functions as a Transcriptional Activator

**Figure 2:**
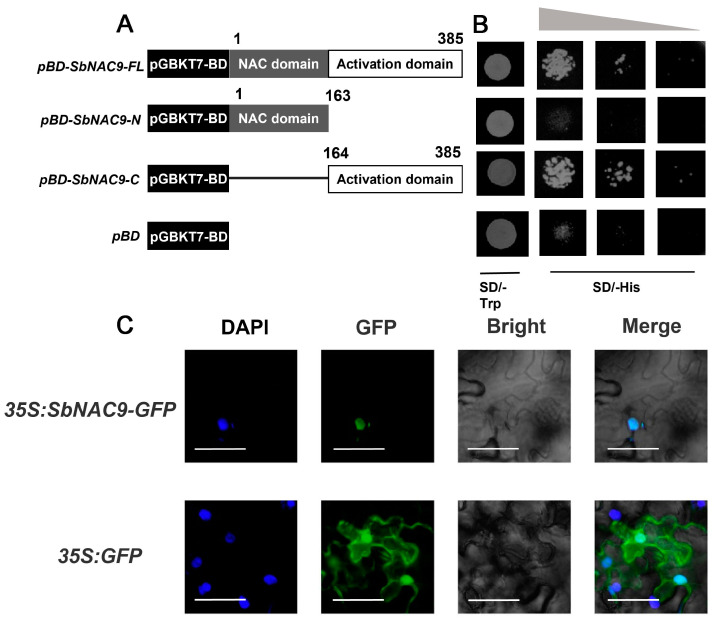
SbNAC9 functions as a transcriptional activator. (**A**) Schematic diagrams of constructs in *SbNAC9* transcriptional activation assay. (**B**) Transcriptional activation of *SbNAC9*. Yeast cells were diluted to 1, 10^−1^, 10^−2^ from the left panel to the right panel. *pBD-SbNAC9-FL*, full-length CDS of *SbNAC9*; *pBD-SbNAC9-N*, N-terminal of *SbNAC9*; *pBD-SbNAC9-C*, C-terminal of *SbNAC9*; and *pBD*, *pGBKT7-BD* vector. *pGBKT7-BD* was used as a negative control. (**C**) Subcellular localization of SbNAC9 in tobacco leaves. DAPI (2-(4-Amidinophenyl)-6-indolecarbamidine dihydrochloride) channel represents the signal of nucleus colored in blue. GFP channel represents the signal of SbNAC9-GFP or GFP colored in green. Merge indicates combination of GFP (green fluorescent protein), DAPI, and Bright together colored in cyan. Bars indicate 50 μm.

*SbNAC9* encodes a 385-amino-acid protein with a 163-amino-acid conserved NAC domain (1-163 aa) harboring five conserved subdomains (a–e) (Figure S2). To test if SbNAC9 had transcription activity, we constructed three vectors as *pBD-SbNAC9-FL* (containing the full length of *SbNAC9*), *pBD-SbNAC9-N* (containing N-terminal of *SbNAC9*), and *pBD-SbNAC9-C* (containing C terminal of *SbNAC9*). In these three vectors, full length or partial coding sequence of *SbNAC9* were fused with GAL4 DNA-binding domain (Figure 2A). Yeast cells transformed with *pBD-SbNAC9-FL* and *pBD-SbNAC9-C*, but not with *pBD-SbNAC9-N*, grew well on the SD/-His medium (Figure 2B), indicating that SbNAC9 may function as a transcriptional activator and its transcriptional activation domain was in the C terminal.

We also analyzed the SbNAC9 protein sequence using an online webtool (https://psort.hgc.jp/) and noticed a nuclear localization signal of PRDRKYP inside the SbNAC9 protein. To examine the subcellular localization of SbNAC9, we checked the protein localization of 35S: SbNAC9-GFP in tobacco leaf cells. A strong GFP signal can be detected in the nucleus (Figure 2C), suggesting that SbNAC9 is a transcriptional activator localized in the nucleus.

### 2.3. Overexpression of SbNAC9 Can Enhance Drought-Stress Tolerance of Sorghum

**Figure 3:**
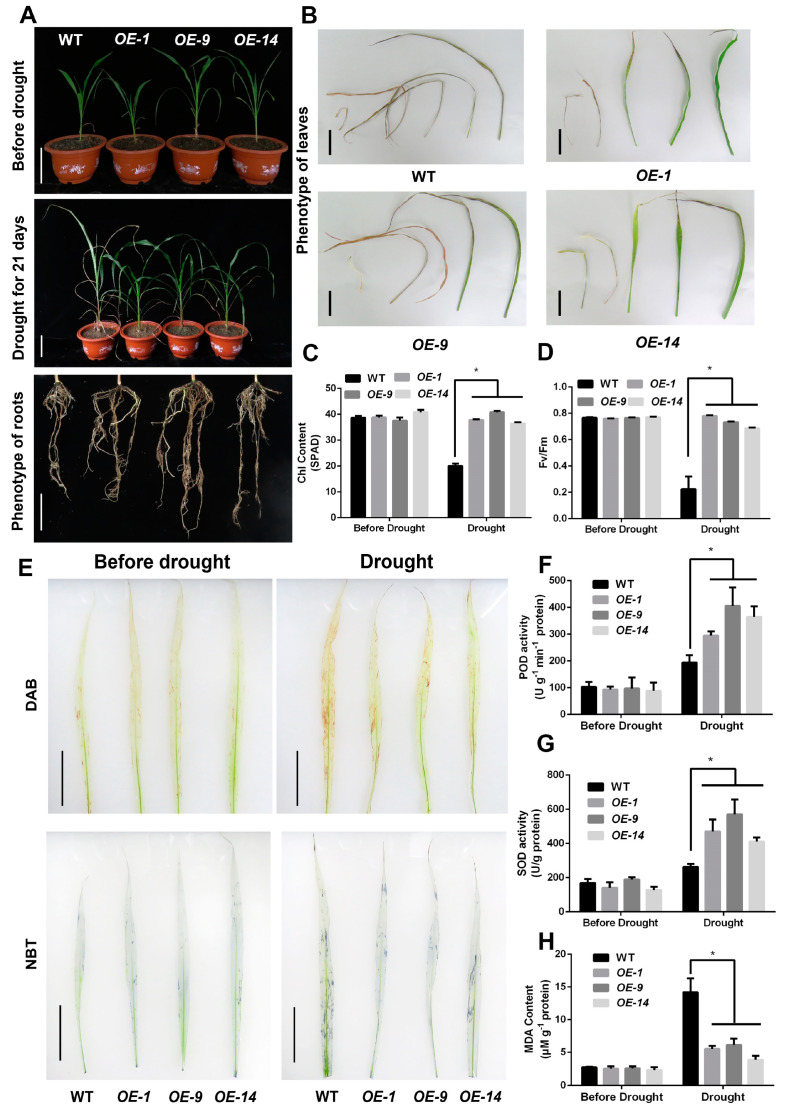
Overexpression of *SbNAC9* enhanced drought-stress tolerance of sorghum. (**A**) Phenotype of sorghum plants under drought-stress treatment. Three-week-old WT and transgenic lines (*OE-1*, *OE-9*, *OE-14*) were treated with water deprivation for 21 days. Bars indicate 10 cm. (**B**) Phenotype of first five leaves of WT and transgenic lines after 21-day drought-stress treatment. Bars indicate 5 cm. (**C**,**D**) Chlorophyll content (**C**) and chlorophyll fluorescence Fv/Fm (**D**) in the fifth leaf of WT and transgenic lines after drought treatment. Error bars indicate SD of three independent experiments. * *p* < 0.05 by Student’s *t-*test. (**E**) DAB and NBT staining of leaves of WT and transgenic sorghum seedlings treated with drought stress for 5 days. Bars indicate 5 cm. (**F**–**H**) POD activity (**F**), SOD activity (**G**), and MDA content (**H**) of WT and transgenic lines after drought-stress treatment. Error bars indicate SD of three independent experiments. * *p* < 0.05 by Student’s *t*-test.

To investigate the role of *SbNAC9* in sorghum under drought stress, we overexpressed *SbNAC9* in sorghum and chose three independent lines with various *SbNAC9* expression levels (*OE-1*, *OE-9*, *OE-14*) for further study (Figure S3). At the six-leaf stage, the selected transgenic lines and wild-type plants were treated with water deprivation for 21 days. The transgenic lines displayed less withered leaves and stronger root architecture than the wild-type plants after drought treatment (Figure 3A,B). Additionally, the transgenic lines showed a lower water loss rate than the wild-type plants (Figure S4).

Chlorophyll content is a vital indicator to assess drought-stress tolerance of plants [30]. Therefore, we measured chlorophyll contents in *SbNAC9* overexpression lines and wild-type plants under normal or continuous drought conditions. The results showed that the transgenic lines had significantly higher chlorophyll content than the wild-type plants under drought treatments (Figure 3C). Consistently, the transgenic lines exhibited an obviously higher Fv/Fm (variable fluorescence/maximal fluorescence) ratio than the wild-type plants (Figure 3D). These results suggested that photosynthesis in sorghum transgenic lines with *SbNAC9* overexpression were less affected by drought stress.

Drought stress triggered oxidative damage to plant cells. Thus, the ability to eliminate the excess accumulation of ROS was also considered a critical index for plant response to drought stress. We compared ROS scavenging activities of transgenic lines with *SbNAC9* overexpression and wild-type plants under drought stress. The DAB and NBT staining assays showed that transgenic lines accumulated less H_2_O_2_ and O^2−^ compared with wild-type (Figure 3E). Meanwhile, transgenic lines had significantly higher POD and SOD activities than the wild-type plants (Figure 3F,G), and the MDA content of transgenic lines was noticeably lower than in wild-type plants (Figure 3H). Taken together, these results showed that overexpression of *SbNAC9* enhanced drought-stress tolerance of sorghum through maintaining relative high photosynthesis, strengthening root architecture, and increasing ROS scavenging ability.

### 2.4. Silencing of SbNAC9 Weakens Drought-Stress Tolerance of Sorghum Seedlings

**Figure 4:**
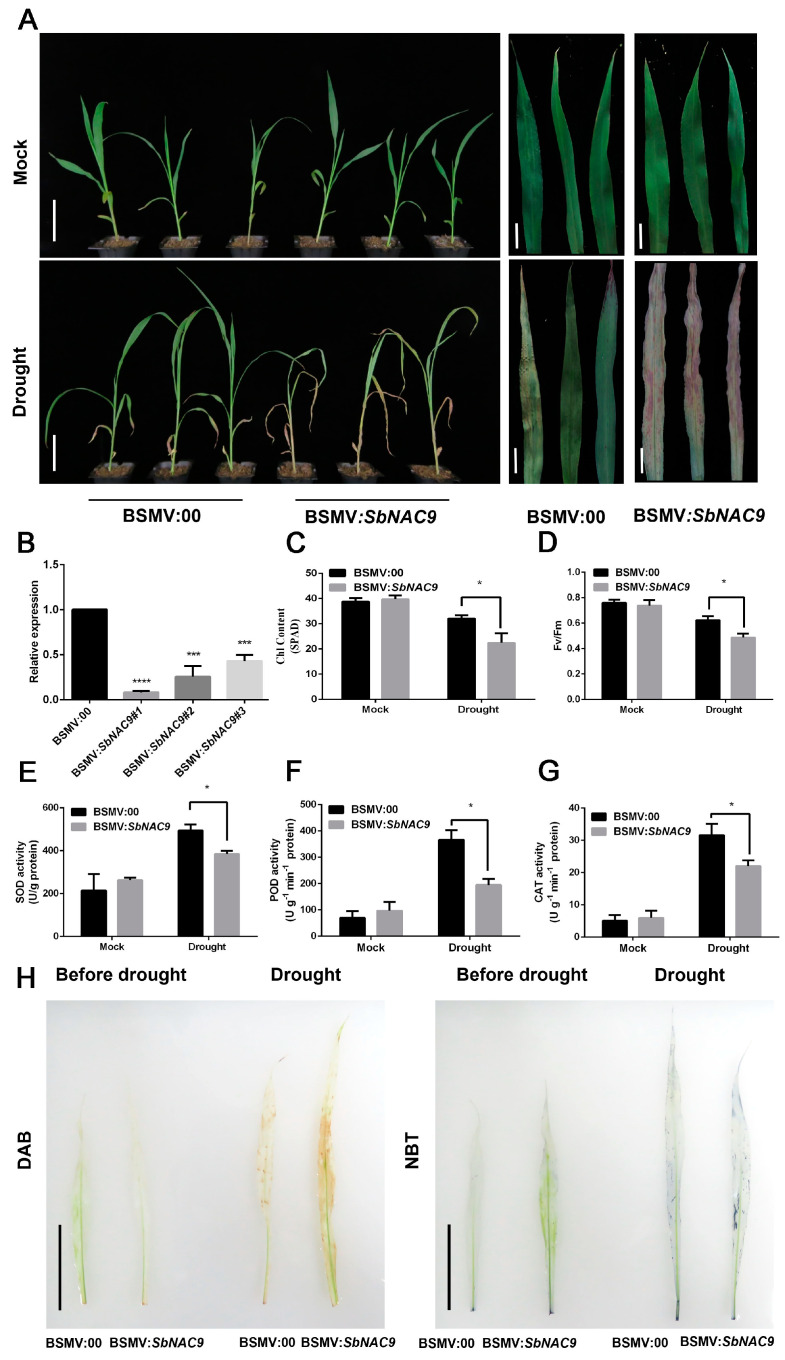
Silenced *SbNAC9* weakened drought-stress tolerance of sorghum. (**A**) Phenotype of sorghum seedlings inoculated with BSMV:00 and BSMV:*SbNAC9* under mock and drought-stress treatments. Bars indicate 6 cm for the whole plants in the left panels and 2 cm for the leaves in the middle and right panels. (**B**) Relative transcript level of *SbNAC9* in sorghum seedlings silenced by VIGS. *SbEIF4A* was used as the internal reference. Error bars indicate SD of three independent experiments. **** *p* < 0.0001 and *** *p* < 0.001 by Student’s *t*-test. (**C**,**D**) Chlorophyll content (**C**) and chlorophyll fluorescence Fv/Fm (**D**) in the third leaf of sorghum seedlings inoculated with BSMV:00 and BSMV:*SbNAC9* under mock or drought-stress treatments. Error bars indicate SD of three independent experiments. * *p* < 0.05 by Student’s *t*-test. (**E**–**G**) SOD (**E**), POD (**F**), and CAT (**G**) activities in sorghum seedlings inoculated with BSMV:00 and BSMV:*SbNAC9* under mock or drought-stress treatments. Error bars indicate SD of three independent experiments. * *p* < 0.05 by Student’s *t*-test. (H) DAB and NBT staining of leaves of sorghum seedlings inoculated with BSMV:00 and BSMV:*SbNAC9* under drought-stress treatment for 5 days. Bars indicate 5 cm.

To further test the role of *SbNAC9* in sorghum under drought stress, we silenced *SbNAC9* in sorghum seedlings by VIGS. We observed phenotypes of plants inoculated with empty virus (BSMV:00) or with BSMV:*SbNAC9* under normal conditions or continuous drought treatments. Sorghum seedlings inoculated with BSMV:*SbNAC9* showed obviously weakened drought stress tolerance with more wilting and chlorosis of leaves than the plants inoculated with empty virus (Figure 4A,B). Consistently, chlorophyll content and Fv/Fm ratio in plants inoculated with BSMV:*SbNAC9* were significantly lower than those in plants inoculated with empty virus (Figure 4C,D). Moreover, the activities of antioxidative enzymes including SOD, POD, and CAT in plants inoculated with BSMV:*SbNAC9* were significantly lower than those in plants inoculated with empty virus under drought-stress treatment (Figure 4E–G). DAB and NBT staining assays also showed that plants inoculated with BSMV:*SbNAC9* had more H_2_O_2_ and O^2−^ than plants inoculated with empty virus (Figure 4H). Together with the traits of *SbNAC9* overexpression lines under drought-stress treatments (Figure 3), all these results suggest that *SbNAC9* may play a vital function in drought-stress tolerance of sorghum.

### 2.5. Heterologous Overexpression of SbNAC9 Enhances Drought Tolerance of Arabidopsis

To further confirm the function of *SbNAC9* in drought-stress response, we also overexpressed *SbNAC9* in Arabidopsis. We treated wild-type Col-0 plants and three independent transgenic lines (*#9*, *#10*, and *#11* with various expression level of *SbNAC9*) with water deprivation for 10 days. After 5 continuous days with rewatering, the transgenic lines showed obviously higher survival rate than wild-type plants (Figure S5A–C). Under drought-stress treatment, transgenic plants showed lower H_2_O_2_ and O^2−^ level by DAB and NBT staining than wild-type (Figure S5D,E). Meanwhile, the antioxidative enzyme activities were higher and MDA content was lower in transgenic lines than those in wild-type plants under drought-stress treatment (Figure S5I).

Next, we examined relative expression of several drought-responsive genes of Arabidopsis including *Dehydration Responsive Element-Binding protein 1A* (*DREB1A*), *Dehydration Responsive Element-Binding protein 2A* (*DREB2A*), and *KIN1* [31,32,33]. We also examined relative expression of a key enzyme gene *9-Cis-Epoxycarotenoid Dioxygenase 3* (*AtNCED3*) involved in biosynthesis of ABA [16], which can regulate stomatal aperture and water loss rate [29]. Compared with wild-type plants, relative expression of these four genes in *SbNAC9* overexpression Arabidopsis was significantly upregulated under drought stress (Figure S6).

Drought stress retarded various developmental processes including root formation. We observed that the roots of transgenic lines were longer than wild-type plants in 10% PEG6000 MS medium (Figure S7). Taken together, heterologous overexpression of *SbNAC9* in Arabidopsis also can enhance plant drought-stress tolerance by reducing water loss, increasing antioxidative activity, and forming elongated roots.

### 2.6. SbNAC9 Directly Activates Expression of SbC5YQ75 and SbNCED3

Many NAC TFs are involved in abiotic stress by regulating downstream stress-induced genes [34]. In Arabidopsis, researchers identified consensus binding sites for two representative NAC proteins, ANAC019 and ANAC092 [35]. SbNAC9 shows higher similarity to ANAC019 by protein sequence alignment (Figure S8). Therefore, we used the putative binding motif (TTNCGTA) of ANAC019 [35] as the putative binding site of SbNAC9. To investigate how *SbNAC9* is involved in drought-stress tolerance of sorghum, we searched for putative downstream targets of SbNAC9 based on the study of differentially expressed genes (DEGs) under sorbitol-simulated osmotic stress in sorghum [27]. We found 16 putative targets of SbNAC9 by searching the 2000-bp promoter sequences (including the proximal promoter and 5′ UTR) upstream of the start codons of these DEGs (Table S2). After prediction of genes’ function and analysis of the positions of SbNAC9-binding motifs, we chose *SbC5YQ75*, *FASCICLIN-LIKE ARABINOGALACTAN 1* (*SbFLA1*), and *SbC5XIY1* as candidates. As *AtNCED3* was significantly upregulated in *SbNAC9* overexpression Arabidopsis, we, therefore, searched the promoters of *SbNCED3* and *SbNCED9*, both of which are *AtNCED3* homologs (Figure S9). We found that both *SbNCED3* and *SbNCED9* promoters contain SbNAC9 binding motifs, with two different bases (TCTCGTG) at -838 bp and one different base (TTGCGTG) at −1126 bp upstream of their transcriptional start sites, respectively. Hence, we finally chose five genes including *SbC5YQ75*, *SbFLA1*, *SbC5XIY1*, *SbNCED3*, and *SbNCED9* for further analysis.

**Figure 5:**
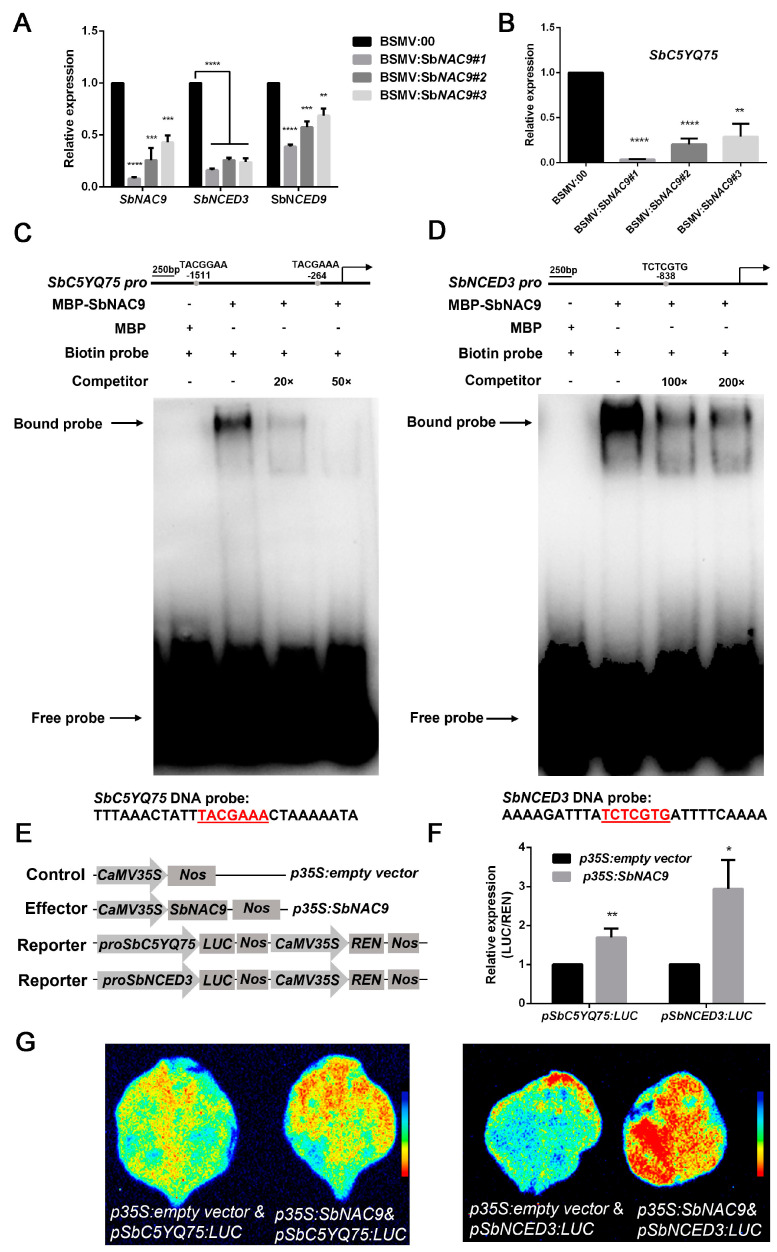
SbNAC9 directly activated the expression of *SbC5YQ75* and *SbNCED3*. (**A**,**B**) Relative transcript level of *SbNAC9*, *SbNCED3*, *SbNCED9* (**A**), and *C5YQ75* (**B**) in the third leaf of sorghum seedlings with silenced *SbNAC9*. *SbEIF4A* was used as the internal reference. Error bars indicate SD of three independent experiments. **** *p* < 0.0001, *** *p* < 0.001, and ** *p* < 0.01 by Student’s *t-*test. (**C**,**D**) EMSA assays of SbNAC9 bound to the promoters of *SbC5YQ75* and *SbNCED3* in vitro. Competition experiments were performed with excessive amounts of unlabeled probes (20× and 50× for *SbC5YQ75* as well as 100× and 200× for *SbNCED3*). Schematic diagrams of putative binding motifs of SbNAC9 on the promoters of *SbC5YQ75* and *SbNCED3* were listed at the top. The motif at −264 bp upstream of transcription start sites of *SbC5YQ75* and the motif at −838 bp upstream of transcription start sites of *SbNCED3* were used for EMSA assays. The sequences of probes were listed at the bottom. (**E**) Schematic diagrams of constructs used in luciferase assays. (**F**) Relative transcript level of LUC/REN activity normalized by *REN* in luciferase assays. Error bars indicate SD of three independent experiments. ** *p* < 0.01 and * *p* < 0.05 by Student’s *t*-test. (**G**) Luciferase assays of SbNAC9 binding on *SbC5YQ75* and *SbNCED3* promoters in tobacco leaves. *pSbC5YQ75:LUC* or *pSbNCED3:LUC* with an empty vector were used as negative control.

We tested the transcript level of these five candidate genes in sorghum seedlings under 20% PEG6000-simulated dehydration-stress treatment and found that expression levels of all the five genes peaked later than *SbNAC9* (Figure S10A). In addition, the five genes also rapidly responded to ABA treatment (Figure S10B). These indicated that these five candidates were the potential targets of SbNAC9 and possibly involved in ABA signaling for drought-stress tolerance. We then tested relative expression of the five genes in sorghum seedlings with silenced *SbNAC9*, and found that expression of *SbC5YQ75*, *SbNCED3*, and *SbNCED9* were significantly downregulated (Figure 5A,B, and Figure S11A). However, due to the obvious upregulation of only *SbC5YQ75*, *SbNCED3*, and *SbNCED9*, but not the other two candidates in sorghum with *SbNAC9*-overexpression (Figure S11), we, therefore, chose *SbC5YQ75*, *SbNCED3*, and *SbNCED9* for further analysis.

To test the direct bindings, we first performed EMSA assays, and found that SbNAC9 directly bound the promoter fragments of *SbC5YQ75* and *SbNCED3*, and the bindings were weakened with increased amounts of competitive probes (Figure 5C,D). However, we could not detect the binding of SbNAC9 to the promoter of *SbNCED9* (Figure S12). Thus, we focused on *SbC5YQ75* and *SbNCED3* for further study. We also performed luciferase assays and found that the relative expression level of LUC/REN was higher in the cotransformed leaves with effector and two individual reporters than the control leaves (Figure 5E–G). All these results indicated that SbNAC9 can directly activate *SbC5YQ75* and *SbNCED3*.

### 2.7. Functions of SbC5YQ75 and SbNCED3 under Drought Stress in Sorghum

**Figure 6:**
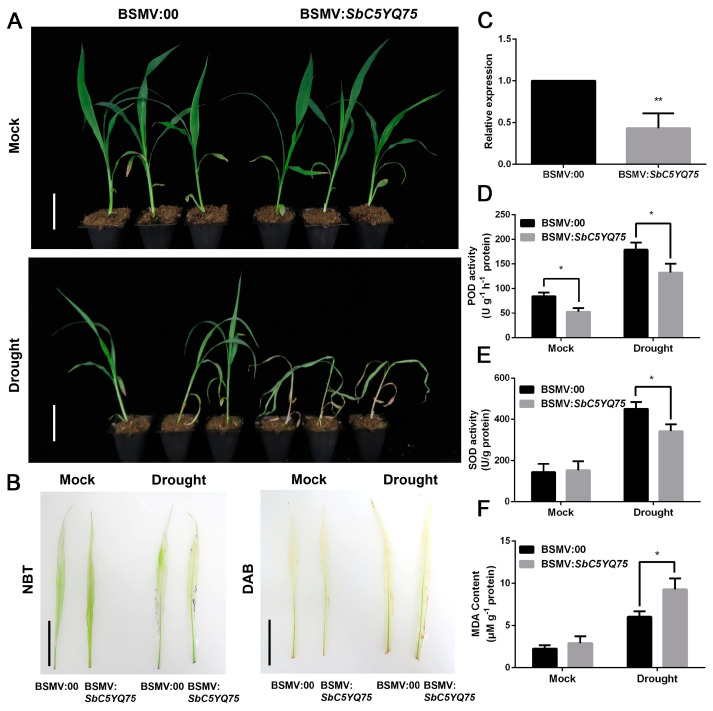
The function of *SbC5YQ75* in response to drought stress in sorghum. (**A**) Phenotype of sorghum seedlings inoculated with BSMV:00 and BSMV:*SbC5YQ75* under mock and drought-stress treatments. Bars indicate 6 cm. (**B**) DAB and NBT staining of leaves of sorghum seedlings inoculated with BSMV:00 and BSMV:*SbC5YQ75* treated with drought stress for 5 days. Bars indicate 5 cm. (**C**) Relative transcript level of *SbC5YQ75* in sorghum seedlings silenced by VIGS. *SbEIF4A* was used as the internal reference. Error bars indicate SD of three independent experiments. ** *p* < 0.01 by Student’s *t*-test. (**D**–**F**) POD and (**D**) SOD (**E**) activities and MDA content (**F**) in sorghum seedlings inoculated with BSMV:00 and BSMV:*SbC5YQ75* under mock or drought-stress treatments. Error bars indicate SD of three independent experiments. * *p* < 0.05 by Student’s *t-*test.

To further explore the functions of the two SbNAC9 targets *SbC5YQ75* and *SbNCED3* under drought stress, we first silenced *SbC5YQ75* by VIGS in the sorghum seedlings. Compared with sorghum seedlings inoculated with empty virus (named BSMV:00), the sorghum seedlings inoculated with BSMV:*SbC5YQ75* (named BSMV:*SbC5YQ75*) showed weakened drought-stress tolerance with more wilted leaves (Figure 6A). The DAB and NBT staining indicated that BSMV:*SbC5YQ75* accumulated more H_2_O_2_ and O^2−^ (Figure 6B,C). Consistently, the activities of antioxidative enzymes including POD and SOD in BSMV:*SbC5YQ75* were more weakened than those in BSMV:00 (Figure 6D,E). Moreover, MDA content in BSMV:*SbC5YQ75* was obviously higher than those in BSMV:00 under drought-stress treatment (Figure 6F). These results indicated that silenced *SbC5YQ75* weakened the plants’ drought-stress tolerance by decreasing ROS scavenging ability in sorghum.

We also silenced *SbNCED3* by VIGS in sorghum seedlings to explore its function in response to drought stress. The sorghum seedlings inoculated with BSMV:*SbNCED3* (named BSMV:*SbNCED3*) exhibited weakened drought-stress tolerance with more wilted leaves than those inoculated with empty virus (named BSMV:00) (Figure S13A). In Arabidopsis, researchers found that AtNCED3 is the key enzyme to promote ABA biosynthesis [16]. To further investigate the role of SbNAC9 and SbNCED3 in ABA signaling, we compared relative ABA content in sorghum seedlings with silenced *SbNAC9* or silenced *SbNCED3* under dehydration treatment. The results showed that the ratio of ABA content of 12 h over that of 0 h after dehydration treatment in sorghum seedlings with silenced *SbNAC9* or silenced *SbNCED3* were both significantly lower than that in BSMV:00 (Figure S13B,D). These suggested that *SbNAC9* and *SbNCED3* can function in ABA signaling, thereby affecting drought-stress resistance of sorghum.

## 3. Discussion

The functions of sorghum NAC TFs which can respond to abiotic stresses remain largely unknown. In this study, we cloned *SbNAC9*, which has 63.22% similarity to *OsNAC5* that can be induced by abiotic stress (Figure S2) [28]. Our results show that *SbNAC9* can be induced by abiotic stresses including low temperature, salt, PEG-simulated, and mannitol-simulated dehydration stress, as well as ABA treatments. Overexpression of *SbNAC9* enhanced drought tolerance of both Arabidopsis and sorghum, while silencing of *SbNAC9* weakened drought tolerance of sorghum. In sorghum, SbNAC9 can directly activate the putative peroxidase gene *SbC5YQ75* and the putative ABA biosynthesis gene *SbNCED3* to enhance plant drought tolerance.

When sensing drought-stress signals, plants evolve various strategies to adapt to water-deficient conditions. Among these strategies, morphological adaptation such as “stay green” of leaves, strengthening of root architecture, and stomatal closure are considered as typical responses of plants [3]. In this study, sorghum transgenic lines with *SbNAC9* overexpression exhibited higher chlorophyll content, higher photosynthetic rate, stronger root architecture, and lower water loss rate than wild-type plants (Figure 3A–D and Figure S4), while seedlings with silenced *SbNAC9* showed opposite traits (Figure 4A–D). These findings suggest that *SbNAC9* plays an essential role by enhancing plant physical adaptation to drought stress.

Drought stress also induces ROS accumulation. The scavenging ability of ROS is vital for plants to respond to drought stress. Our previous study showed that *SbNAC2* overexpression in Arabidopsis has higher antioxidative enzyme activities under multiple abiotic stresses than wild-type plants [26]. In this study, we showed that SbNAC9 is both capable and required for ROS scavenging in sorghum ((Figure 3E–G and (Figure 4E–H). To investigate the molecular function of SbNAC9, we analyzed two putative peroxidases, *SbC5YQ75* and *SbC5XIY1*. Given SbNAC9 as a transcription activator (Figure 2A,B), we tested the transcript level of these two genes in sorghum seedlings with silenced *SbNAC9*, and only *SbC5YQ75* was downregulated ((Figure 5B and Figure S11A). Our EMSA assay and luciferase assays confirmed the direct activation of *SbC5YQ75* by SbNAC9 (Figure 5C–F). Moreover, sorghum seedlings with silenced *SbC5YQ75* showed weakened drought-stress tolerance with decreased antioxidative enzyme activities under drought stress (Figure 6). Taken together, SbNAC9 may enhance the ROS scavenging ability of sorghum by directly activating *SbC5YQ75* expression.

ABA level quickly elevates when plants sense drought-stress signals [36]. Previous studies revealed that many TFs are involved in ABA signaling pathway [16]. In Arabidopsis, the NAC transcription factor ATAF1 can bind to the TTGCGTA motif in the promoter of *NCED3* to regulate ABA biosynthesis [37]. In rice, WRKY5 functions as a negative regulator by binding to the abiotic stress-related gene *OsMYB2*, which can enhance ABA-induced drought tolerance [38]. The mutants *oswrky5–2* and *oswrky5–3* showed enhanced drought-stress tolerance and higher sensitivity to ABA. In this study, *SbNAC9* was rapidly upregulated under ABA treatment (Figure S10B). Additionally, the transcript level of *NCED3* involved in ABA biosynthesis was upregulated under drought stress in Arabidopsis transgenic lines overexpressing *SbNAC9* (Figure S6). In sorghum, we showed that SbNAC9 can directly induce *SbNCED3* (Figure 5C–F), which may be potentially involved in ABA biosynthesis. Moreover, after dehydration treatment, we found that the relative ABA content in sorghum seedlings with silenced *SbNCED3* was much lower than that in plants inoculated with empty virus. Sorghum seedlings with silenced *SbNAC9* showed similar results (Figure S13B–D). Thus, *SbNAC9* can be induced by ABA, and in turn, SbNAC9 may possibly promote ABA biosynthesis by directly activating the putative ABA biosynthesis gene *SbNCED3*, thereby forming a positive feedback loop for drought-stress response in sorghum.

Recent reports provided details on the molecular and structural study about NAC TFs. For example, transgenic tobacco overexpressing the NAC domain of GhNAC4 exhibits higher tolerance to stress and higher sensitivity to ABA compared with lines overexpressing GhNAC4 transactivation domain [39]. In maize, ZmMPK5 can phosphate ZmNAC49 on the Thr-26 by increasing the binding capability of ZmNAC49 to *ZmSOD3* and, thus, enhances the oxidative stress tolerance of maize [40]. Natural antisense transcript also participates in drought stress as well. Cis-NATZmNAC48 represses the expression of *ZmNAC48* and affects stomatal movement to alter drought-stress tolerance of maize [41]. Whether SbNAC9 can be similarly regulated like these NAC TFs remains to be uncovered.

**Figure 7:**
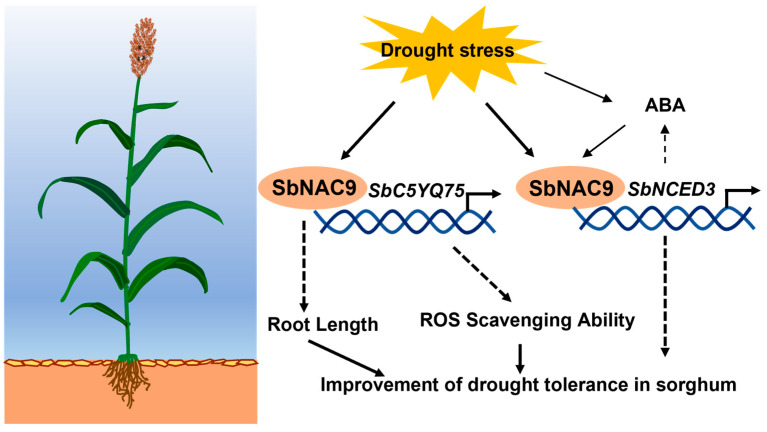
Model for SbNAC9 function in drought-stress tolerance of sorghum. Drought stress induces *SbNAC9* expression. SbNAC9 directly activates the expression of *SbC5YQ75* and *SbNCED3* by binding to their promoters. In addition, SbNCED3 may potentially promote biosynthesis of ABA, which can induce *SbNAC9* expression to form a feedback regulation. Additionally, *SbNAC9* overexpression enhances drought-stress tolerance of sorghum through altering root architecture and increasing ROS scavenging ability.

In summary, we proposed a model of how SbNAC9 enhances drought-stress tolerance in sorghum (Figure 7). Drought stress can induce *SbNAC9* expression. Thereby, SbNAC9 directly activates the expression of *SbC5YQ75* and *SbNCED3* by binding to their promoters. In addition, SbNCED3 may potentially promote biosynthesis of ABA, which can induce *SbNAC9* expression to form a feedback regulation. Additionally, *SbNAC9* overexpression enhances drought-stress tolerance of sorghum through strengthening root architecture and increasing ROS scavenging ability.

## 4. Materials and Methods

### 4.1. Plant Materials and Growth Condition

Tx430, a nontannin genotype, was used for sorghum transformation. The transgenic lines were grown at 28 °C under 12 h light/12 h dark photoperiod at 60% humidity for seed harvest. The sorghum genotype BTx623 was used for the other assays. The sorghum seedlings used in the study were grown at 25 °C under 16 h light/ 8 h dark photoperiod at 60% humidity. Transformation of Arabidopsis was performed on Columbia wild-type (Col-0). Tobacco (*Nicotiana tabacum*) was used for the subcellular localization assay and luciferase assay. Arabidopsis and tobacco used in the study were grown at 24 °C under 16 h light/ 8 h dark photoperiod at 60% humidity.

### 4.2. RNA Extraction and Quantitative Real-Time PCR (qRT-PCR) Assay

Total RNA extraction, cDNA synthesis, and qRT-PCR were carried out as previously described [42]. *Actin2* and *SbEIF4A* were used as internal controls in Arabidopsis and sorghum, respectively. The primers used in qRT-PCR are listed in Table S3.

### 4.3. Phytohormone and Abiotic Stress Treatments

The roots of sorghum seedlings at four-leaf stage were subjected to 150 μM GA, 150 μM ABA, 200 mM NaCl, 200 mM mannitol, 20% PEG6000 treatments or kept at 4 °C, and the fourth leaves were collected for qRT-PCR. For drought-stress treatments, sorghum seedlings overexpressing *SbNAC9* at six-leaf stage were treated with water deprivation for 21 days. An amount of 15 sorghum plants each of WT and transgenic lines were used for control and drought-stress treatments. The sorghum seedlings with VIGS-mediated silencing of *SbNAC9*, *SbC5YQ75*, and *SbNCED3* were treated with water deprivation for 7 days on the sixth day after inoculation with virus. Then, 12 sorghum seedlings each with BSMV:00 or silenced *SbNAC9*, *SbC5YQ75*, or *SbNCED3* were used for control and drought-stress treatments. Three biological replicates were performed in drought-stress treatments. Three-week-old Arabidopsis transgenic plants heterologously overexpressing *SbNAC9* and WT plants were treated with water deprivation for 10 days.

### 4.4. In Situ Hybridization Assay

The sorghum seeds used for the in situ hybridization assay were sterilized by 30% sodium hypochlorite for 10 min, repeated three times, and then the seeds were washed by sterilized water five times. The sterilized seeds were put on the filter paper to absorb water completely. The seeds were grown on the MS medium for three days. The root tips were collected and soaked in 20% PEG solution for 4 h and 6 h, and then embedded into paraffin for in situ hybridization or put into liquid nitrogen for qRT-PCR. The in situ hybridization assay was performed as previously described [42]. The primer sequences used for probes are listed in Table S3.

### 4.5. Transactivation Assay

The pGBKT7 (Clontech) constructs containing the full-length N-terminal and C-terminal coding sequences of *SbNAC9* were transformed into yeast strain AH109. Empty pGBKT7 was used as the negative control. SD/-Trp medium was used for selecting the successful transformants. SD/-His medium was used to test the transactivation activity. The primers are listed in Table S3.

### 4.6. Subcellular Localization Assay

The coding sequence of *SbNAC9* (without stop codon) was amplified and fused with GFP coding sequence within the vector pGreen to generate *35S:SbNAC9-GFP*. *Agrobacterium* strain GV3101 with pSoup containing *35S:SbNAC9-GFP* or *35S:GFP* was transformed into three-week-old tobacco leaves. After two days of infiltration, the leaves were harvested and stained by 10 μg/mL DAPI for one hour and then GFP fluorescence was observed via Olympus (BX53) microscope. The primers are listed in Table S3.

### 4.7. Transformation of Arabidopsis and Sorghum

We used the pGreen vector harboring *35S:SbNAC9-GFP* for plant transformation. Agrobacterium strain GV3101 with pSoup was used. The method of Arabidopsis transformation was carried out as previously described [43]. Sorghum transformation was performed according to a previous protocol [44].

### 4.8. Measurement of Chlorophyll Content

Chlorophyll contents in the fifth leaves of WT and transgenic lines overexpressing *SbNAC9*, as well as in the third leaves of sorghum seedlings with silenced *SbNAC9*, were measured by a chlorophyll meter SPAD-502 PLUS (Konika Minolta, Tokyo, Japan).

### 4.9. Measurement of Chlorophyll Fv/Fm

We selected the fifth leaf of sorghum seedlings overexpressing *SbNAC9* and WT, as well as the third leaf of sorghum seedlings with silenced *SbNAC9* by VIGS, for the measurement of chlorophyll fluorescence Fv/Fm. The measurement was performed as previously described [45].

### 4.10. Measurement of Antioxidative Enzyme Activities and MDA Content

Rosette leaves from transgenic Arabidopsis, the fifth leaf from transgenic sorghum seedlings overexpressing *SbNAC9*, and the fourth leaf from sorghum seedlings with silenced *SbNAC9* and *SbC5YQ75* were used for the measurement of antioxidative enzyme activities and MDA content. The SOD, CAT, and POD activity measurement and MDA content measurement were performed as previously described [12].

### 4.11. Diaminobenzidine (DAB) and Nitroblue Tetrazolium (NBT) Staining Assays

Rosette leaves of transgenic Arabidopsis and leaves of sorghum seedlings were collected into 1 mg/mL DAB staining solution (prepared in 50 mM phosphate buffer pH3.8). The leaves were placed at 25 °C for 6 h and decolored by 90% ethanol in boiling water bath. The decolored leaves were placed in petri dish filled with water for taking photos. The NBT staining was conducted as previously described [40].

### 4.12. Virus-Induced Gene Silencing (VIGS) Assay

*Barley stripe mosaic virus* (BSMV)-based vectors α, β, and γ were used for VIGS. The target fragments of *SbNAC9*, *SbNCED3*, and *SbC5YQ75* were predicted by SGN VIGS Tool (https://vigs.solgenomics.net/). The predicted target fragments were inserted into γ vector. The primers are listed in Table S3. The α, β, γ, γ-*SbNAC9*, γ-*SbNCED3*, and γ*-SbC5YQ75* were transformed into EHA105. The VIGS assay was performed as previously described [26]. Sorghum seedlings used for VIGS assays were grown under normal conditions for 5 days after being inoculated with virus, and then treated with water deprivation for 7 days.

### 4.13. Water Loss Rate Assay

The sixth leaves of sorghum seedlings at six-leaf stage were used for measurement of water loss rate. The assays were performed as previously described [23]. Briefly, the detached leaves were placed in a growth chamber at 25 °C. Fresh weight of detached leaves was recorded every 15 min from when the leaves were collected from sorghum.

### 4.14. Electrophoretic Mobility Shift (EMSA) Assay

The coding sequence of *SbNAC9* was amplified and introduced into the vector pMAL-c5G. The construct was transformed into Transetta competent cells (TransGen Biotech, Beijing, China) to acquire MBP-SbNAC9 fusion protein. The EMSA assays were conducted as previously described [42]. The primers used for this assay are listed in Table S3.

### 4.15. Luciferase Assay

The 2000-bp promoter fragments of *SbC5YQ75* and *SbNCED3* were cloned into pGreenII-0800-LUC (luciferase) which contains a separated 35S Renilla (REN) luciferase reporter gene for normalization. Then, *35S:SbNAC9* was used as an effector. Luciferase assays were carried out as described previously [46].

### 4.16. Measurement of ABA Content in Sorghum

Two-week-old sorghum seedlings were used to measure ABA content under dehydration treatment. Before treatment, roots of sorghum seedlings were soaked in water for 16 h. Then, sorghum seedlings were transferred from water to 50 mL empty tubes for 12 h dehydration treatment. To measure ABA content, 100 mg tissue from the fourth leaves of each group were collected into liquid nitrogen. The frozen ground leaves were then mixed with PBS buffer. After centrifugation, the supernatant was used for ABA measurement according to the instruction of ABA ELISA Kit (MBE21031; MALLBIO Biological Technology, Nanjing, China).

### 4.17. Statistical Analysis

Statistical analyses were performed by GraphPad Prism (GraphPad Software Inc., San Diego, CA, USA). Experimental data were analyzed by one-way ANOVA test with ** *p* < 0.01 and * *p* < 0.05 for Figure S4 and by Student’s *t-*test with * *p* < 0.05, ** *p* < 0.01, *** *p* < 0.001, and **** *p* < 0.0001 for the other figures.

### 4.18. Gene Accession Number

SbNAC9 (SOBIC.005G064600.2) was obtained from Phytozome13 of JGI Genome Portal website (https://genome.jgi.doe.gov/portal/, accessed on 9 January 2018). All gene information in Table S2 as well as *SbNCED3* (Sb01g013520) and *SbNCED9* (Sb02g003230) were obtained from PlantGDB (http://www.plantgdb.org/, accessed on 2 February 2020). All gene information of Arabidopsis was obtained from TAIR (https://www.arabidopsis.org/, accessed on 3 October 2020) according to the accession numbers as follows: *DREB1A* (AT4G25480), *DREB2A* (AT5G05410), *KIN1* (AT5G15960), and *NCED3* (AT3G14440).

## Data Availability

The data presented in this study are available in the article or in the supplementary material.

## Supplementary Materials

The supporting information can be downloaded at: https://www.mdpi.com/article/10.3390/ijms24032401/s1.

## Abbreviations

ABA	Abscisic acid
BSMV	Barley stripe mosaic virus
CAT	Catalase
DAB	Diaminobenzidine
Fv/Fm	Fluorescence/maximal fluorescence
GA	Gibberellin
GFP	Green fluorescent protein
LUC	Luciferase
MDA	Malondialdehyde
NBT	Nitroblue tetrazolium
PEG	Polyethylene glycol
POD	Peroxidase
REN	Renilla
ROS	Reactive oxygen species
SOD	Superoxide dismutase
TFs	Transcription factors
VIGS	Virus-induced gene silencing
