# Supplementary material for: SbNAC9 Improves Drought Tolerance by Enhancing Scavenging Ability of Reactive Oxygen Species and Activating Stress-Responsive Genes of Sorghum
Source: Int J Mol Sci. 2023 Jan 26;24(3):2401. doi: 10.3390/ijms24032401 (PMC9917103; doi:10.3390/ijms24032401)
Supplement: Supplementary file 1 [file ijms-24-02401-s001.zip › ijms-2025397-supplementary.pdf]

Supplementary Materials

Table S1. The cis-acting elements in 2000-bp promoter of *SbNAC9*

Name	Function	Number
ABRE	cis-acting element involved in the abscisic acid responsiveness	12
A-Box	cis-acting regulatory element	6
ARE	cis-acting regulatory element essential for the anaerobic induction	1
CGTCA-motif	cis-acting regulatory element involved in the MeJA-responsiveness	14
GC-motif	enhancer-like element involved in anoxic specific inducibility	3
TATC-box	cis-acting element involved in gibberellin-responsiveness	1
TATA-box	core promoter element around -30 of transcription start	13
RY-element	cis-acting regulatory element involved in seed-specific regulation	1
TGACG-motif	cis-acting regulatory element involved in the MeJA-responsiveness	14
Box 4	part of a conserved DNA module involved in light responsiveness	1
CAAT-box	common cis-acting element in promoter and enhancer regions	13
CAT-box	cis-acting regulatory element related to meristem expression	3
G-Box	cis-acting regulatory element involved in light responsiveness	10
GA-motif	part of a light responsive element	1
L-box	part of a light responsive element	1
MBS	MYB binding site	1
Sp1	light responsive element	3

The cis-acting elements were predicted by Plant CARE database (http://bioin formatics.psb.ugent.be/webtools/plant care/html/)

Table S2. Screened candidate target genes of SbNAC9

Accession Number	Annotation	Gene function	Putative Binding sites
Sb01g016600.1	A0A1B6QJR7	peroxidase, putative	711
Sb01g033390.1	C5WXN2	serine-type carboxypeptidase	196
Sb06g001690.1	C5YC92	germin-like protein, putative	127
Sb02g042870.1	A0A1B6QGB6	peroxidase, putative	1057
Sb06g018740.1	A0A1B6PLA9	gamma-glutamyltransferase/ glutathione gamma-glutamylcysteinyltransferase	606
Sb03g030340.1	C5XFX7	(1-4)-beta-mannan endohydrolase, putative	1006
Sb02g012920.1	C5X6P7	copper ion binding / electron carrier	925
Sb10g000470.1	C5Z240	copper ion binding / oxidoreductase	949
Sb04g024440.1	C5XWE5	glycerophosphodiester phosphodiesterase/ kinase	1507
Sb03g013200.1	C5XIY0	peroxidase, putative	1932
Sb01g018490.1	C5WXD7	secretory protein, putative	175
Sb02g000840.1	C5X780	plastocyanin-like domain-containing protein	605
Sb08g000990.1	C5YQ75	peroxidase, putative	264, 1511
Sb09g004700.1	C5Z0P5	FASCICLIN-LIKE ARAB INOGALACTAN 1	684

Accession Number	Annotation	Gene function	Putative Binding sites
Sb03g013210.1	C5XIY1	peroxidase, putative	182
Sb06g021290.1	A0A1B6PLT5	unknown protein	81

The genes underlined were selected as candidate genes of SbNAC9. *Sb09g004700.1* was the homologue gene of *AtFLA1* in sorghum. Gene accession numbers were obtained from PlantGDB (http://www.plantgdb.org/).

Table S3. Primers and probes used for qRT-PCR, DNA constructs and EMSA

Name	Forward	Reverse
**qRT-PCR**		
SbEIF4A	CAACTTTGTCACCCGCGATGA	TCCAGAAACCTTAGCAGCCCA
ACTIN2	CTGGAATGGTGAAGGCTGG	TTGGATACTTCAGAGTGAG
SbNAC9	CCCGTCGCCATCAAGAAG	GCACCCAGTCATCCAGCCT
DREB2A	TTAGGCAAAGGATTTGGGGTA	CAGAAGCAGCTTCTTGAGCAG
DREB1A	CGGTAAGTGGGTTTGTGAGGT	TCCAAGCCGAGTCAGCGA
KIN1	CCTTCCAAGCCGGTCAGAC	TGCCGCATCCGATACACTC
NCED3	GACAAGAACAAGGTCGCAAGA	CCAGAGATGGAAGCAGAAGCA
SbNCED3	CAACGAGTCCGACGAGCG	GGCGTACCGTGTCTTGCG

SbNCED9	CACCAAGCCGTTCCTCAAGT	GGGATGATGGCGTGGTTCT
C5YQ75	TCGAGGACGGCGAGCTT	GCAGCGTGCTAAGCCAATG
FLA1	GACGGCGAAGGCGAAGA	GTGTCATCCTTGCTCTCGGC
C5XIY1	CCTGACGGGCAAGCTCTTC	CGGTGAGGTTCTTCCTCCTG
REN	ATAACTGGTCCGCAGTGGTG	TAAGAAGAGGCCGCGTTACC
LUC	ATTACACCCGAGGGGGATGA	CTCTCACACACAGTTCGCCT
**DNA constructs**		
Tblunt-SbNAC9-*in situ*	GCCCTCAGGCTGGATGACT	TCCACGCCACGGAGTTGT
35S:SbNAC9-GFP	GCTACGCGTCTCGAGATGGACTGCGGTGGCG	TCCGGCGCCGGGCCCGAACGGCTTGTGGAGGTACG
MBP-SbNAC9	GCGGCACACTACGTACATATGATGGACTGCGGTGGCG	ACCTGCAGGGAATTCGAACGGCTTGTGGAGGTACG
pBD-SbNAC9 (FL)	GAGGAGGACCTGCATATGATGGACTGCGGTGGCG	ACGGATCCCCGGGAATTCCTAGAACGGCTTGTGGAGGTA
pBD-SbNAC9 (N)	GAGGAGGACCTGCATATGATGGACTGCGGTGGCG	ACGGATCCCCGGGAATTCTTGTTGTAGATCCGGCACAG
pBD-SbNAC9 (C)	GAGGAGGACCTGCATATGAAGGGCGTGATCGAGCG	ACGGATCCCCGGGAATTCCTAGAACGGCTTGTGGAGGTA

pDEST22-SbNAC9	GCAGGCTCCGCGGCCGCATGGACTGCGGTGGCG	GTGAAGGGGGCGGCCGCCTAGAACGGCTTGTGGAGGTA
pHisi-1-SbC5YQ75	AATTCTATTTACGAAACTAAACTATTTACGAAACTAAACTATTTACGAAACTAAAT	CTAGATTTAGTTTCGTAAATAGTTTAGTTTCGTAAATAGTTTAGTTTCGTAAATAG
pHisi-1-SbNCED3	AATTCATTTATCTCGTGATTTTATTTATCTCGTGATTTTATTTATCTCGTGATTTT	CTAGAAAATCACGAGATAAATAAAATCACGAGATAAATAAAATCACGAGATAAATG
p35S:C5YQ75:LUC	CTGCAGCCCGGGGGATCCGTTTCAGGATTTACGGAATA	GGCGTCTTCCATGCGGCCGCCTCGTACACAAGCTAGCTAGTCT
p35S:SbNCED3:LUC	CTGCAGCCCGGGGGATCCCCGTGTGTTCGTTCCTCTTC	GGCGTCTTCCATGCGGCCGCCACGGTGGCTGAATTGG
γ-SbNAC9-VIGS	CTTCCGTTTCTAAGGAAGTTTAACCGACGGGCCACCATAA	TTTTAACCACCACCACCGTGACTCGGACCCAGAGCGC
γ-SbC5YQ75-VIGS	CTTCCGTTTCTAAGGAAGTTTAATTGAAGGTCTAGAGGCGCCA	TTTTAACCACCACCACCGTAAAAGGACATGGTCGCGCT
γ-SbNCED3-VIGS	CTTCCGTTTCTAAGGAAGTTTAACGGGCTTGTGGAACGG	TTTTAACCACCACCACCGTCCCGCCCACCTCTGTTT
**Probes used for EMSA**		
C5YQ75-Bio	TTTAAACTATTTACGAAACTAAAAATA	TATTTTTAGTTTCGTAAATAGTTTAAA

SbNCED3-Bio	AAAAGATTTATCTCGTGATTTTCAAAA	TTTTGAAAATCACGAGATAAATCTTTT
SbNCED9-Bio	TGTCACTGACTTGCGTGCGGACGGACA	TGTCCGTCCGCACGCAAGTCAGTGACA
C5YQ75-Com	TTTAAACTATTTACGAAACTAAAAATA	TATTTTTAGTTTCGTAAATAGTTTAAA
SbNCED3-Com	AAAAGATTTATCTCGTGATTTTCAAAA	TTTTGAAAATCACGAGATAAATCTTTT
SbNCED9- Com	TGTCACTGACTTGCGTGCGGACGGACA	TGTCCGTCCGCACGCAAGTCAGTGACA

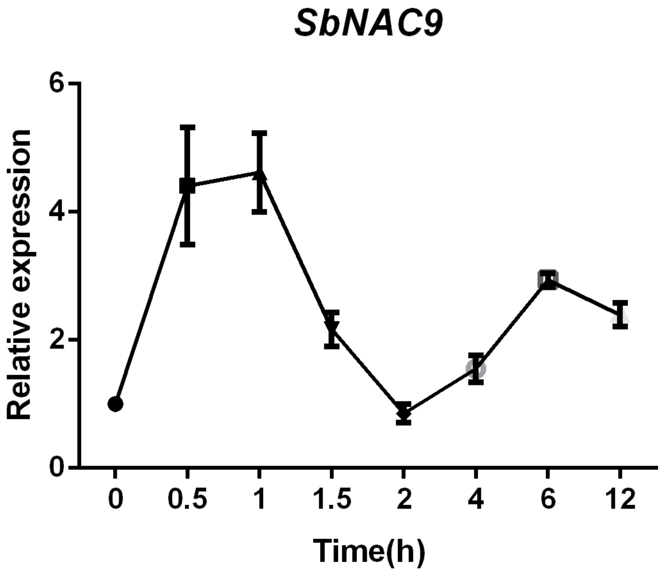

Figure S1. Time course transcript level of *SbNAC9* under ABA treatment. *SbNAC9* expression in sorghum seedlings at four-leaf stage treated with 150 μM ABA. *SbEIF4A* was used as the internal reference. Error bars indicate SD of three independent experiments.

```
OsNAC5     ........MECGG.ALQLPPGFRFHPTDDELVMYYLCRKCGGLPLAAPVIAEVDLYKFNP    51
SbNAC9     ........MDCGG.ALQLPPGFRFHPTDDELVMYYLLRKCGGLPLAAPVIAEVDLYKFDP    51
SbNAC2     ........MSGGGQDLQLPPGFRFHPTDEELVMHYLCRRCAGLPIAVPIIAEIDLYKFDP    52
SbSNAC1    MGLPVMRRERDAEAELNLPPGFRFHPTDDELVEHYLCRKAAGQRLPVPIIAEVDLYKFDP    60
Consensus                      l lppgfrfhptd elv  yl r   g     p iae dlykf p
                                           a                        b

OsNAC5     WDLPERAMGGEKEWYFFSPRDRKYPNGQRPNRAAGTGYWKATGADKPVGSP.RAVAIKKA   110
SbNAC9     WQLPEKAYGGEKEWYFFSPRDRKYPNGSRPNRAAGTGYWKATGADKPVGSP.RPVAIKKA   110
SbNAC2     WQLPRMALYGEKEWYFFSPRDRKYPNGSRPNRAAGSGYWKATGADKPVGTP.KPLAIKKA   111
SbSNAC1    WDLPERALFGVREWYFFTPRDRKYPNGSRPNRAAGNGYWKATGADKPVAPRGRTLGIKKA   120
Consensus  w lp   a   g   ewyff prdrkypng rpnraag gywkatgadkpv        ikka
                                        c

OsNAC5     IVFYAGKPPKGVKTNWIMHEYRLADVDRSAAARKLSKSSHNALRLDDWVLCRIYNKKGVI   170
SbNAC9     IVFYAGKPPKGVKTNWIMHEYRLADVDRSAAARKKTN...NALRLDDWVLCRIYNKKGVI   167
SbNAC2     IVFYAGKAPKGEKTNWIMHEYRLADVDRSARKK.......NSLRLDDWVLCRIYNKKGGL   164
SbSNAC1    IVFYAGKAPRGVKTDWIMHEYRLADAGRAAASKK......GSLRLDDWVLCRIYNKKNEW   174
Consensus  lvfyagk p g kt wimheyrlad   r a          lrlddwvlcr ynkk
                     d                                  e

OsNAC5     PRYDTVDAG......EIVKPAAAAAAAKGGRIGGGGG....AAAMKVELSDYG.FYDQE.   218
SbNAC9     PRYDTVDDDADDAVAEIVKPAPASRNNPRGTASGRGGAAAAAAPMKVEFPEYGGYYDYED   227
SbNAC2     PKPSAVAGG.......DHKPMFAAAAVSSPPE.........QKPFVAAPGGLPPFPDLA.   207
SbSNAC1    PKMQLGKES.....AAGVGTAKEEAMDMTTSHSHS......HSQSHSHSHSWGETRTPE.   222
Consensus  e

OsNAC5     ....PESEMLCFDRSGSADRDSMPRLHTDSSGSEHVLSPSPSPDDFPGGGD.........   265
SbNAC9     LEATPSAGMLCFDRP.SAAVPTAPASAPAPAPGPALSSPPPAADSDPERDDDSNNSVAWT   286
SbNAC2     .........AYYDRP....SDSMPRLHADSSCSECVLSPEQLACDREVQSQ.........   245
SbSNAC1    .......SEIVDNDP.FPELDSFPAFQDPAAAMMMVPKKECVDDGSAAANA.........   265
Consensus                             p

OsNAC5     ..HDYAESQPSGGCGG.......................WP...GVDWAAVGDDGFVID   296
SbNAC9     MHHTHAHTDNYSSCGSEHVLSPSPDLPDRDHAESQSAGLWWPVGVGGDWAAA.EDGFMVD   345
SbNAC2     .....PKIS....................................EWERTFASDFVNP   262
SbSNAC1    .....AKSSD................................LPVDLSYDDIQGMYSG   286
Consensus

OsNAC5     S......SLFELPSPAAFSRAAGDGAAFGDMFT..YLQKP                       328
SbNAC9     VDVDDGSSLFGPPSPG.LPFAFVDAAAFGDMLAS.YLHKP                       383
SbNAC2     AG.....SMLDFVVGG.....HAGDPLLQDILM..YWGKP                       290
SbSNAC1    L......DMLPPPGEDFFSSLFASPRVKGNQPAGAAGLGP                       320
Consensus                                         p
```

Figure S2. Multiple alignments of protein sequences of three SbNAC TFs and OsNAC5. Identical amino acids of SbNAC9, OsNAC5, SbNAC2 and SbSNAC1 protein sequences are shown in black color. The conserved NAC domain is divided into five subdomains (a–e) which are underlined.

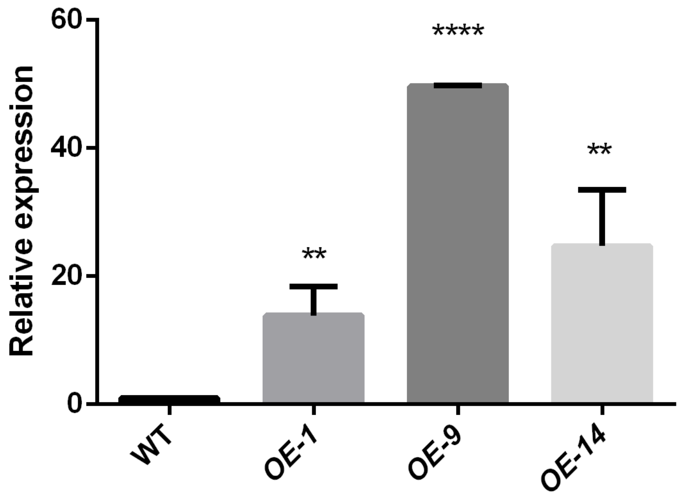

Figure S3. Relative transcript levels of *SbNAC9* in transgenic lines of sorghum. Samples were extracted from sorghum seedlings at six-leaf stage. *SbEIF4A* was used as the internal control. Error bars indicate SD of three independent experiments. ****P<0.0001 and **P<0.01 by Student's t-test.

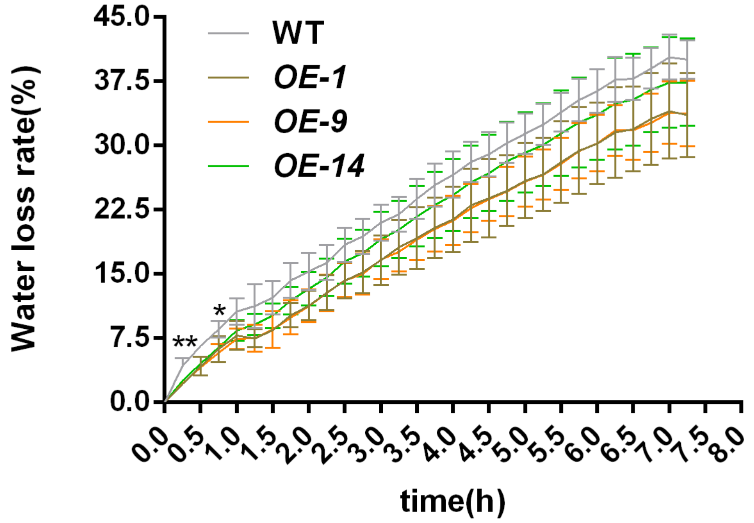

Figure S4. Water loss rate of WT and transgenic lines under normal conditions in sorghum. The detached sixth leaves of these lines were collected to measure water loss rate. Error bars indicate SD of three independent experiments. **P<0.01 and *P<0.05 by one-way ANOVA.

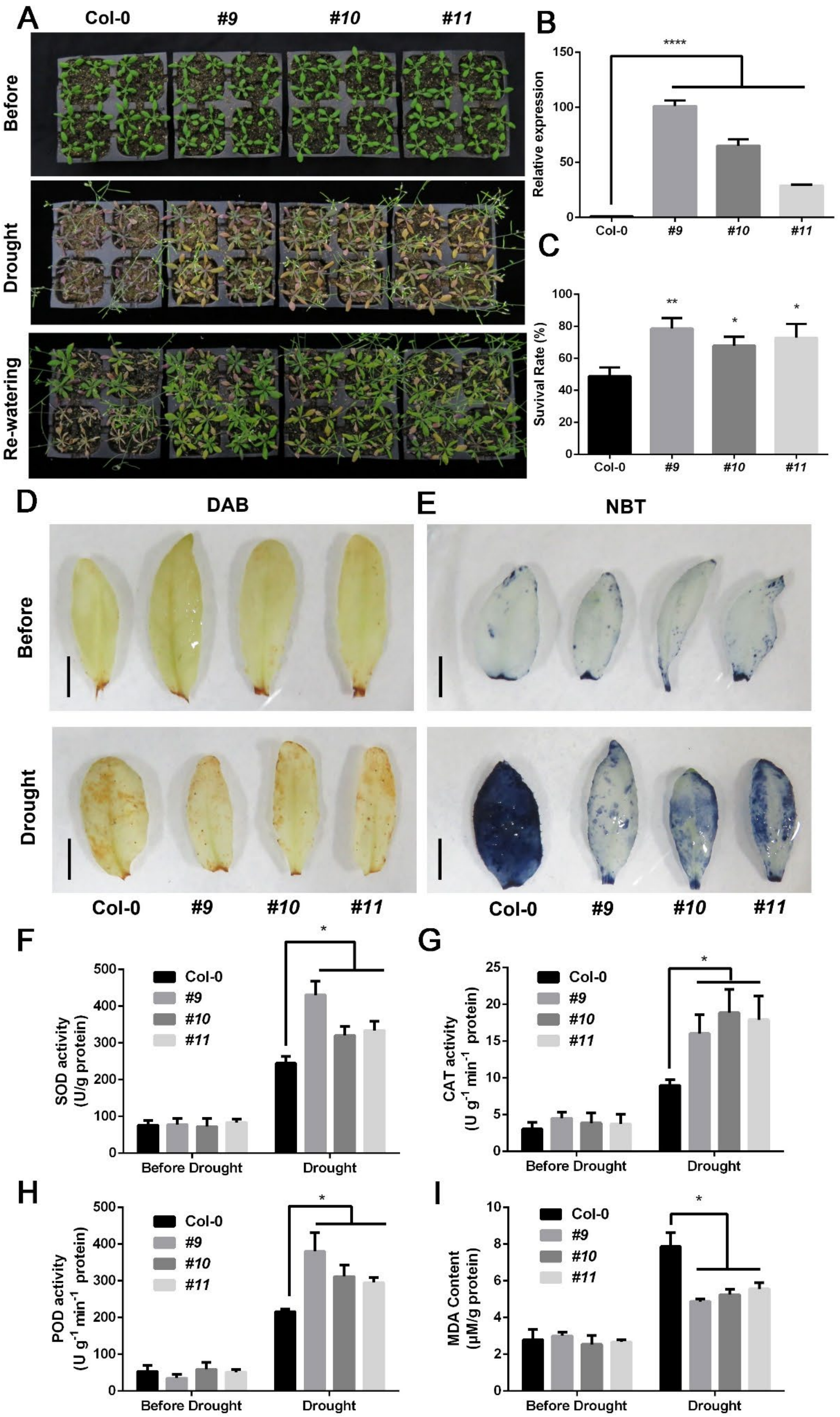

A
Col-0
#9
#10
#11
Before
Drought
Re-watering
B
Relative expression
****
Col-0
#9
#10
#11
C
Suvival Rate (%)
**
*
*
Col-0
#9
#10
#11
D
DAB
E
NBT
Before
Drought
Col-0
#9
#10
#11
Col-0
#9
#10
#11
F
SOD activity (U/g protein)
Col-0
#9
#10
#11
Before Drought
Drought
G
CAT activity (U g-1 min-1 protein)
Col-0
#9
#10
#11
Before Drought
Drought
H
POD activity (U g-1 min-1 protein)
Col-0
#9
#10
#11
Before Drought
Drought
I
MDA Content (μM/g protein)
Col-0
#9
#10
#11
Before Drought
Drought

Figure S5. Heterologous over-expression of *SbNAC9* enhanced drought tolerance of Arabidopsis. (A) Plant phenotype under drought stress treatment. Three-week-old WT (Col-0) and transgenic lines (*#9*, *#10*, *#11*) were treated with water deprivation for 10 days and re-watering for 5 days. (B) Relative transcript levels of *SbNAC9* in WT and transgenic lines of Arabidopsis. *Actin2* was used as the internal reference. Error bars indicate SD of three independent experiments. ****P<0.0001 by Student's t-test. (C) Survival rate of WT and transgenic lines in (A). Error bars indicate SD of more than 30 individual plants. **P<0.01 and *P<0.05 by Student's t-test. (D-E) DAB (D) and NBT (E) staining of rosette leaves from WT and transgenic lines treated with drought stress for 5 days. Bars indicate 1 cm. (F-I) SOD (F), CAT (G) and POD (H) activities, and MDA content (I) of rosette leaves from WT and transgenic lines after drought stress treatment. Error bars indicate SD of three independent experiments. *P<0.05 by Student's t-test.

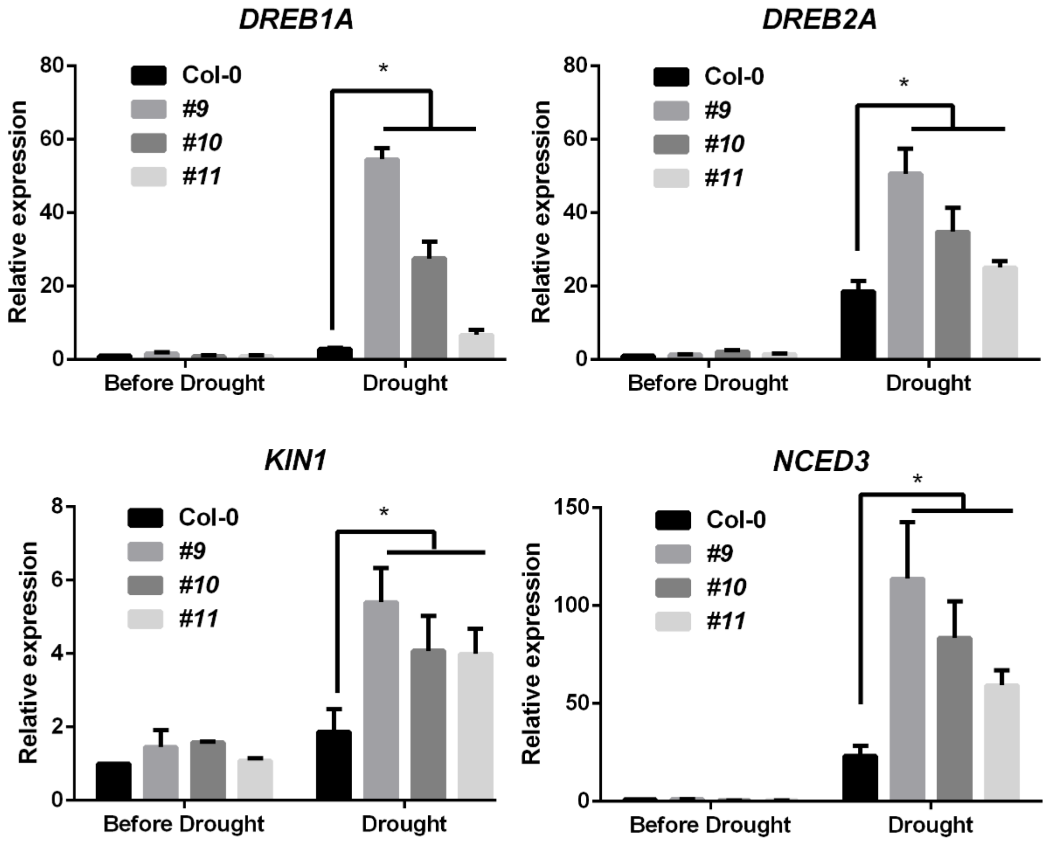

Figure S6. Relative transcript level of drought-inducible genes and genes involved in ABA signaling. *Actin2* was used as the internal reference. Error bars indicate SD of three independent experiments. *P<0.05 by Student's t-test.

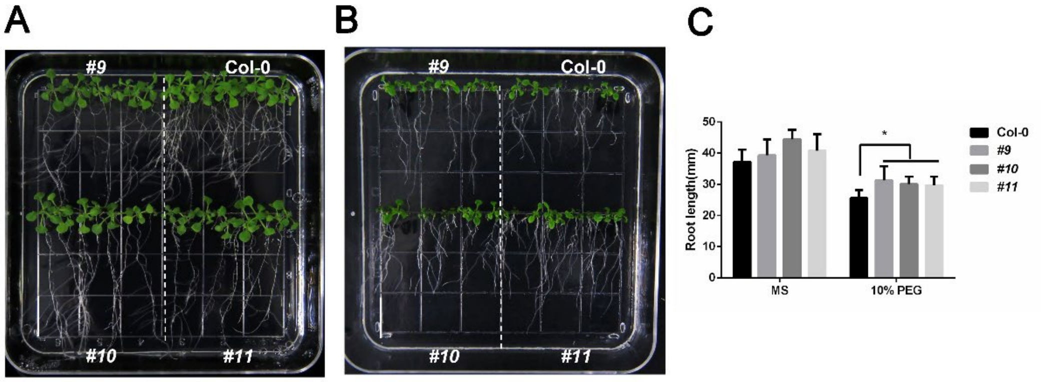

Figure S7. Root length of transgenic Arabidopsis plants with heterologous over-expression of *SbNAC9*. Seedlings grown on MS media (A) and MS medium with 10% PEG (B). Bars indicate 1.5 cm. (C) Root length in (A) and (B). Error bars indicate SD from ten individual plants. *P<0.05 by Student's t-test

A

```
SbNAC9     .....MDCGGALQLPPGFRFHPTDDELVMYYLLRKCGGLPLAAPVIAEVDLYKFDPWQLP    55
AtNAC019   MGIQETDPLTQLSLPPGFRFYPTDEELMVQYLCRKAAGYDFSLQLIAEIDLYKFDPWVLP    60
Consensus        d    l lppgfrf ptd el    yl rk g       iae dlykfdpw lp

SbNAC9     EKAYGGEKEWYFFSPRDRKYPNGSRPNRAAGTGYWKATGADKPVGSP.RPVAIKKALVFY   114
AtNAC019   NKALFGEKEWYFFSPRDRKYPNGSRPNRVAGSGYWKATGTDKIISTEGQRVGIKKALVFY   120
Consensus   ka  gekewyffsprdrkypngsrpnr ag gywkatg dk       v ikkalvfy

SbNAC9     AGKPPKGVKTNWIMHEYRLADVDRSAAARKKTNNALRLDDWVLCRIYNKKGVIERYDTVD   174
AtNAC019   IGKAPKGTKTNWIMHEYRLIEPSR.......RNGSTKLDDWVLCRIYKKQ.........S   164
Consensus   gk pkg ktnwimheyrl    r         n   lddwvlcriy k

SbNAC9     DDADDAVAEDVKPAPASRNNPRGTASGRGGAAAAAAPMKVEFPEYGGYYDYEDLEATPSA   234
AtNAC019   SAQKQVYDNGIANAREFSNN......GTSSTTSSSSHFEDVLDSFHQEIDNRNFQFSNPN   218
Consensus               a    nn      g                      d

SbNAC9     GMLCFDRPSAAVPTAPASAPAPAPGPALSSPPPAADSDPERDDDSNNSVAWTMHHTHAHT   294
AtNAC019   RISSL.RPDLTEQKTGFHGLADTSNFDWASFAGNVEHNNSVPELGMSHVVPNLEYNCGYL   277
Consensus        rp            a        s                  v

SbNAC9     DNYSSCGSEHVLSPSPDLPDRDHAESQSAGLWWPVGVGGDWAAAEDGFMVDVDVDDGSSL   354
AtNAC019   KTEEEVESSHGFNNSGELAQKGYGVDSFG...YSGQVGG......FGFM...........   317
Consensus         s h    s  l                  vgg       gfm

SbNAC9     FGPPSPGLPFARVDAAAFGDMLASYLHKP                                  383
AtNAC019   .............................                                  317
Consensus
```

B

```
SbNAC9     ...........MDCGGALQLPPGFRFHPTDDELVMYYLLRKCGGLPLAAPVIAEVDLYKF    49
AtNAC092   MDYEASRIVEMVEDEEHIDLPPGFRFHPTDEELITHYLKPKVFNTFFSATAIGEVDLNKI    60
Consensus                     lppgfrfhptd el   yl  k       a  i evdl k

SbNAC9     DPWQLPEKAYGGEKEWYFFSPRDRKYPNGSRPNRAAGTGYWKATGADKPVGSPR.PVAIK   108
AtNAC092   EPWDLPWKAKMGEKEWYFFCVRDRKYPTGLRTNRATEAGYWKATGKDKEIFKGKSLVGMK   120
Consensus   pw lp ka  gekewyff  rdrkyp g r nra   gywkatg dk        v  k

SbNAC9     KALVFYAGKPPKGVKTNWIMHEYRLADVDRSAAARKKTNNALRLDDWVLCRIYNKKGVIE   168
AtNAC092   KTLVFYKGRAPKGVKTNWVMHEYRLEGKYCIENLPQTAKN.....EWVICRVFQKRADGT   175
Consensus  k lvfy g  pkgvktnw mheyrl              n      wv cr   k

SbNAC9     RYDTVDDDADDAVAEDVKPAPASRNNPRGTASGRGGAAAAAAPMKVEFPEYGGYYDYEDL   228
AtNAC092   KVPMSMLDPHINRMEPAGLPSLMDCSQRDSFTGSSSHVTCFSDQETED.......KRLVH   228
Consensus         d      e            r    g             e

SbNAC9     EATPSAGMLCFDRPSAAVPTAPASAPAPAPGPALSSPPPAADSDPERDDDSNNSVAWTMH   288
AtNAC092   ESKDGFGSLFYSDPLFLQDNYSLMKLLLDGQETQFSGKPFDGRDSSGTEELDCVWNF...   285
Consensus  e     g l    p                     s  p    d

SbNAC9     HTHAHTDNYSSCGSEHVLSPSPDLPDRDHAESQSAGLWWPVGVGGDWAAAEDGFMVDVDV   348
AtNAC092   ............................................................   285
Consensus

SbNAC9     DDGSSLFGPPSPGLPFARVDAAAFGDMLASYLHKP                            383
AtNAC092   ...................................                            285
Consensus
```

Figure S8. Protein sequences alignment of SbNAC9, AtNAC019 and AtNAC092.

Identical amino acids of between SbNAC9 and AtNAC019 (A), or between SbNAC9 and AtNAC092 (B) protein sequences are shown in black color. The sequence alignment was conducted by DNAMAN.

```
AtNCED3   ...MASFTATAAVSGRWLGGNHTQPPLSSSQSSDLSYCSSLPMASRVTRKLNVSSALHTP   57
SbNCED3   MQSLAPPTSVSIHRQHLPASGSSRARASNSVRFSPRAVSSVPRATAPAERLQAP..FHKP   58
SbNCED9   ...............................MASSISVPAPPAAPATAAPSQAR..PKKP   27
Consensus                                          p a               p

AtNCED3   PALHFPKQSSNSPAIVVKPKAKES.....NTKQMNLFQRAAAAALDAAEGFLVS..HEKL  110
SbNCED3   GAADLPAQSKKPATAIAVPRHAAAPRKAGGKKQLNFFQRAAAAALDAFEEGFVANVLERP  118
SbNCED9   SQLNLTTGKTKTP.VPARPMRAVP........EWNPLQRLAAAALDAVEEGLVAGFLERA   78
Consensus                   p               n  qr aaaalda e   v    e

AtNCED3   HPLPKTADPSVQIAGNFAPVNEQPVRRNLPVVGKLPDSIKGVYVRNGANPLHEPVTGHHF  170
SbNCED3   HGLPSTADPAVQIAGNFAPVGERPPVRELPVSGRIPPFINGVYARNGANPCFDPVAGHHL  178
SbNCED9   HPLPRTADPAVQIAGNYAPVGERPPTGDLPVSGRVPACLDGVYVRNGANPLHAPRAGHHL  138
Consensus h lp tadp vqiagn apv e p    lpv g  p    gvy rnganp   p  ghh

AtNCED3   FDGDGMVHAVKFEHGSAS.YACRFTQTNRFVQERQLGRPVFPKAIGELHGHTGIARLMLF  229
SbNCED3   FDGDGMVHALRIRNGVAESYACRFTETARLTQERAIGRPVFPKAIGELHGHSGIARLALF  238
SbNCED9   FDGDGMLHAVRLRAGRAESYACRFTETARLRQERAIGRP....AIGELHGHSGVARLLLF  194
Consensus fdgdgm ha     g a  yacrft t r  qer  grp    aigelhgh g arl lf

AtNCED3   YARAAAGIVDPAHGTGVANAGLVYFNGRLLAMSEDDLPYQVQITPNGDLKTVGRFDFDGQ  289
SbNCED3   YARAACGLVDPSAGTGVANAGLVYFNGHLLAMSEDDLPYHVRVADDGDLETVGRYDFDGQ  298
SbNCED9   GARSLCGLLDASRGVGVANAGLVYHDNRLLAMSEDDLPYHVRVTADGDLETVGRYDFGGQ  254
Consensus  ar   g  d   g gvanaglvy    llamseddlpy v     gdl tvgr df gq

AtNCED3   LESTMIAHPKVDPESGELFALSYDVVSKPYLKYFRFSPDGTKSPDVEIQLDQPTMMHDFA  349
SbNCED3   LGCAMIAHPKLDPVTGELHALSYDVIKKPYLKYFYFRPDGTKSDDVEIPLDQPTMIHDFA  358
SbNCED9   LDTAMIAHPKLDPATGELFSLSYNVVTKPFLKYFYFTADGRKSPDVEIPVDAPTMMHDFA  314
Consensus l   miahpk dp  gel  lsy v  kp lkyf f  dg ks dvei  d ptm hdfa

AtNCED3   ITENFVVVPDQQVVFKLPEMIRGGSPVVYDKNKVARFGILDKYAEDSSNIKWIDAPDCFC  409
SbNCED3   ITENFVVVPDHQVVFKLQEMLRGGSPVVLDKEKTSRFGVLPKHASDASEMVWVDVPDCFC  418
SbNCED9   VTENHAIIPDQQIVFKLQEMLLGGSPVVYDKNKTARFGVLPKRATDASRLQWVEVPDCFC  374
Consensus  ten    pd q vfkl em  ggspvv dk k  rfg l k a d s   w   pdcfc

AtNCED3   FHLWNAWEEPETDEVVVIGSCMTPPDSIFNE...SDENLKSVLSEIRLNLKTGESTRRPI  466
SbNCED3   FHLWNAWEDEATGEVVVIGSCMTPADSIFNE...SDERLESVLTEIRLDTRTGRSTRRAV  475
SbNCED9   FHLWNAWEDDATGDIVVIGSCMTPADAVFNESAAGEESFRSVLSEIRLDPRTGTSSRRAV  434
Consensus fhlwnawe   t   vvigscmtp d  fne     e   svl eirl   tg s rr

AtNCED3   ISNEDQQVNLEAGMVNRNMLGRKTKFAYLALAEPWPKVSGFAKVDLTTGEVKKHLYGDNR  526
SbNCED3   LP.PSQQVNLEVGMVNRNLLGRKTRYAYLAVAEPWPKVSGFAKVDLETGELTKFEYGEGR  534
SbNCED9   LS.DADQVNLEAGMVNRQLLGRKTRYAYLAIAEPWPKVSGFAKVDLEAGTVEKFIYGEGR  493
Consensus       qvnle gmvnr  lgrkt  ayla aepwpkvsgfakvdl  g   k  yg  r

AtNCED3   YGGEPLFLPGE.....GGEEDEGYILCFVVV.............................  552
SbNCED3   FGGEPCFVPMDPSAAHPRGEDDGYVLTFVHDERAGTSELLVVNAADMRLEATVQLPSRVP  594
SbNCED9   YGGEPCFVPRP.DAPAGAAEDDGYVLCYVHDEGRGASEMLVVNARDMRAEAAVKLPGRVP  552
Consensus  ggep f p          ed gy l  v

AtNCED3   ................                                              552
SbNCED3   FGFHGTFITGKELEAQ                                              610
SbNCED9   YGLHGTFIVGEELQRQ                                              568
Consensus
```

Figure S9. Multiple alignments of protein sequences of AtNCED3, SbNCED3 and SbNCED9. Identical amino acids of AtNCED3, SbNCED3 and SbNCED9 protein sequences are shown in black color.

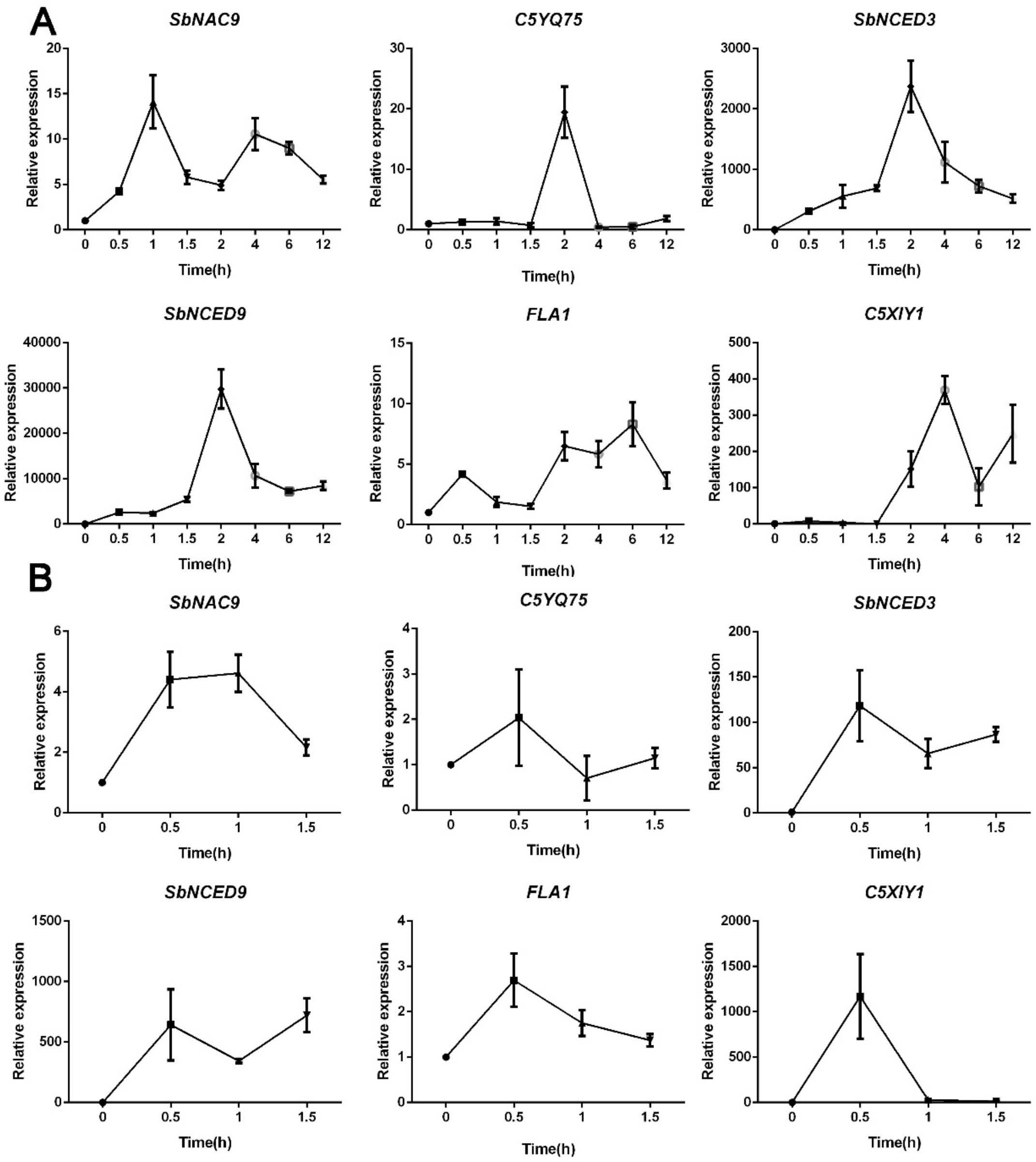

Figure S10. Time course transcript level of candidate genes of SbNAC9 under PEG-simulated dehydration treatment and ABA treatment. (A-B) Relative transcript level of *SbNAC9* and its candidate genes in sorghum seedlings at four-leaf stage subjected to 20% PEG treatment (A) and 150 μM ABA treatment (B). *SbEIF4A* was used as the internal reference. Error bars indicate SD of three independent experiments.

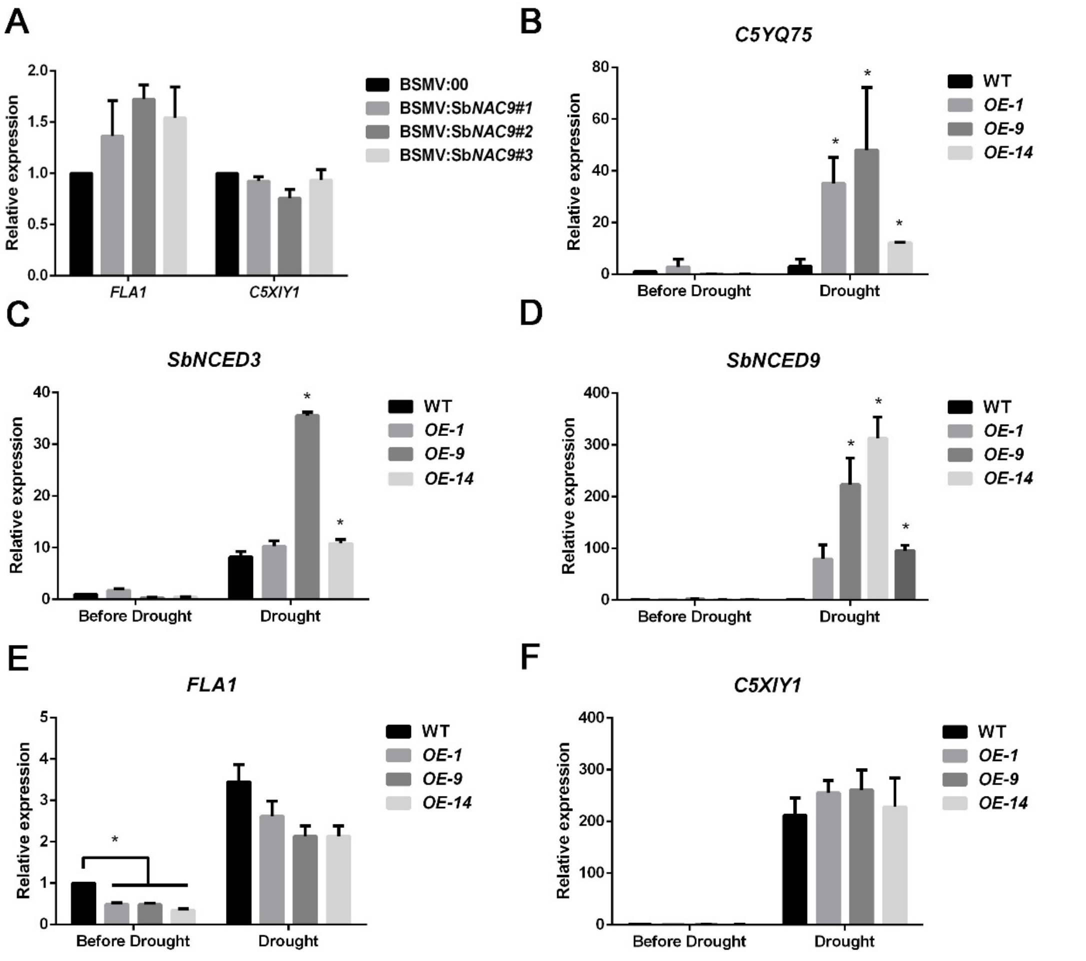

Figure S11. Relative transcript level of candidate genes of SbNAC9 in sorghum seedlings with silenced *SbNAC9* and in transgenic lines of *SbNAC9*-overexpression. Relative transcript level of *FLA1* and *C5XIY1* in the third leaf of sorghum seedlings with silenced *SbNAC9* (A). Relative transcript level of *C5YQ75* (B), *SbNCED3* (C), *SbNCED9* (D). *FLA1* (E), *C5XIY1* (F) in transgenic lines of *SbNAC9*-overexpression. Samples were extracted from sixth leaves of sorghum. *SbEIF4A* was used as the internal control. Error bars indicate SD of three independent experiments. *P<0.05 by Student's t-test.

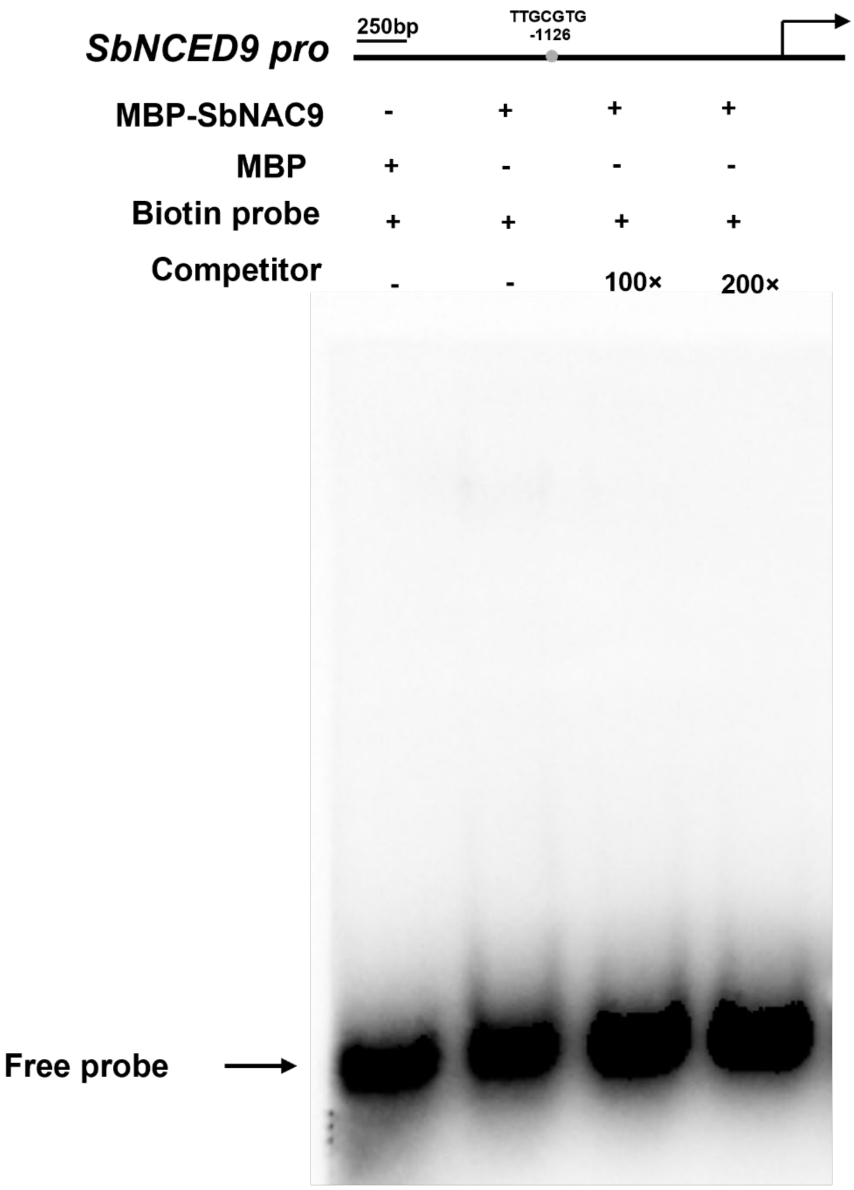

***SbNCED9* DNA probe:**
TGTCACTGACTTGCGTGCGGACGGACA

Figure S12. EMSA assay showing SbNAC9 could not bind to the promoter of *SbNCED9* in vitro. Competition experiments were performed with excessive amounts of unlabeled probes (100× and 200× for *SbNCED9*). Schematic diagrams of putative binding motifs of SbNAC9 on the promoter of *SbNCED9* was listed at the top. The motif at -1126 bp upstream of transcription start site of *SbNCED9* was used for EMSA assays. The sequence of probe was listed at the bottom.

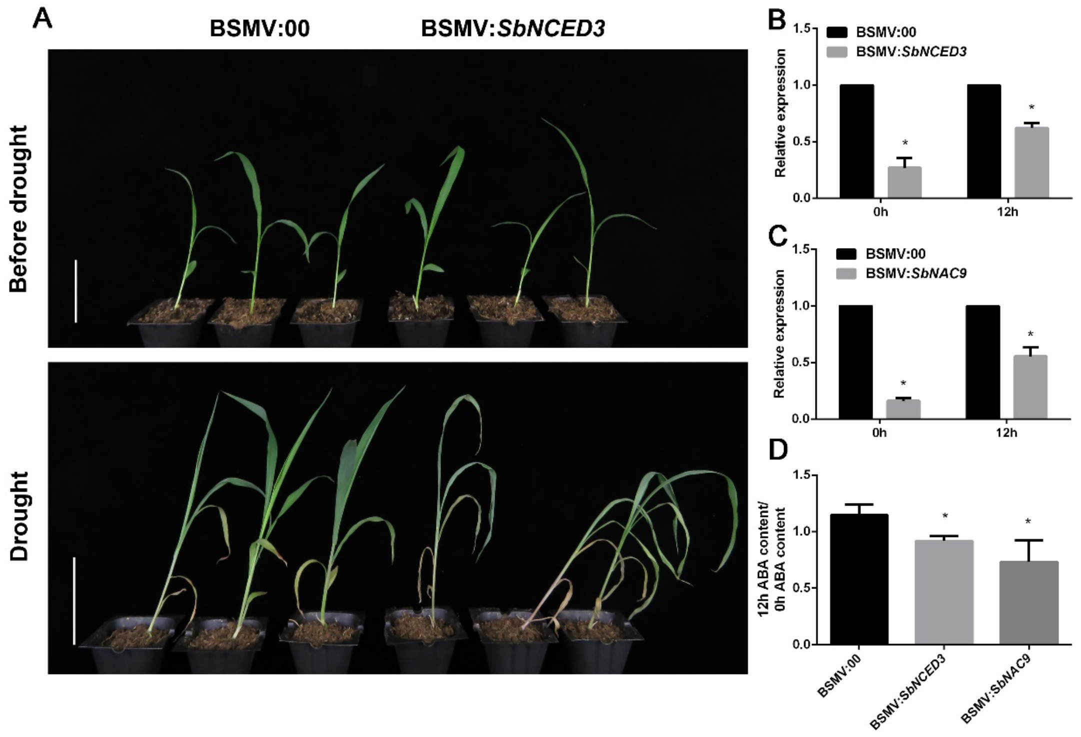

Figure S13. The function of *SbNCED3* in response to drought stress in sorghum. (A) Phenotype of sorghum seedlings inoculated with BSMV:00 and BSMV:*SbNCED3* under drought stress treatment. Bars indicate 6 cm. (B-C) Relative transcript level of *SbNCED3* and *SbNAC9* in sorghum seedlings silenced by VIGS. *SbEIF4A* was used as the internal reference. Error bars indicate SD of three independent experiments. *P<0.05 by Student's t-test. (D) The ratio of ABA content in sorghum seedlings with 12-h dehydration treatment over that with 0-h dehydration treatment, in plants inoculated with BSMV:00, or in plants with silenced *SbNCED3* or *SbNAC9*. Error bars indicate SD of three independent experiments. *P<0.05 by Student's t-test.
